# Genome-wide analysis of the RpoN regulon in *Geobacter sulfurreducens*

**DOI:** 10.1186/1471-2164-10-331

**Published:** 2009-07-22

**Authors:** Ching Leang, Julia Krushkal, Toshiyuki Ueki, Marko Puljic, Jun Sun, Katy Juárez, Cinthia Núñez, Gemma Reguera, Raymond DiDonato, Bradley Postier, Ronald M Adkins, Derek R Lovley

**Affiliations:** 1 Department of Microbiology, University of Massachusetts, Amherst, MA 01003, USA; 2 Department of Preventive Medicine, the University of Tennessee Health Science Center, Memphis, TN 38163, USA; 3 Genomatica, Inc, 10520 Wateridge Circle, San Diego, CA 92121, USA; 4 Departamento de Ingeniería Celular y Biocatálisis, Instituto de Biotecnología, Universidad Nacional Autónoma de México, AP 510-3, Cuernavaca, Mor. 62250, México; 5 Departamento de Microbiología Molecular, Instituto de Biotecnología, Universidad Nacional Autónoma de México, AP 510-3, Cuernavaca, Mor. 62250, México; 6 Department of Pediatrics, the University of Tennessee Health Science Center, Memphis, TN 38163, USA; 7 Department of Microbiology and Molecular Genetics, Michigan State University, East Lansing, MI 48823, USA; 8 Department of Biology, Washington University in St Louis, One Brookings Dr, Campus Box 1137, St Louis, MO 63130, USA

## Abstract

**Background:**

The role of the RNA polymerase sigma factor RpoN in regulation of gene expression in *Geobacter sulfurreducens *was investigated to better understand transcriptional regulatory networks as part of an effort to develop regulatory modules for genome-scale *in silico *models, which can predict the physiological responses of *Geobacter *species during groundwater bioremediation or electricity production.

**Results:**

An *rpoN *deletion mutant could not be obtained under all conditions tested. In order to investigate the regulon of the *G. sulfurreducens *RpoN, an RpoN over-expression strain was made in which an extra copy of the *rpoN *gene was under the control of a *taclac *promoter. Combining both the microarray transcriptome analysis and the computational prediction revealed that the *G. sulfurreducens *RpoN controls genes involved in a wide range of cellular functions. Most importantly, RpoN controls the expression of the *dcuB *gene encoding the fumarate/succinate exchanger, which is essential for cell growth with fumarate as the terminal electron acceptor in *G. sulfurreducens*. RpoN also controls genes, which encode enzymes for both pathways of ammonia assimilation that is predicted to be essential under all growth conditions in *G. sulfurreducens*. Other genes that were identified as part of the RpoN regulon using either the computational prediction or the microarray transcriptome analysis included genes involved in flagella biosynthesis, pili biosynthesis and genes involved in central metabolism enzymes and cytochromes involved in extracellular electron transfer to Fe(III), which are known to be important for growth in subsurface environment or electricity production in microbial fuel cells. The consensus sequence for the predicted RpoN-regulated promoter elements is TTGGCACGGTTTTTGCT.

**Conclusion:**

The *G. sulfurreducens *RpoN is an essential sigma factor and a global regulator involved in a complex transcriptional network controlling a variety of cellular processes.

## Background

RpoN (σ^54 ^or sigma 54) is a subunit of the RNA polymerase and plays a critical role in the regulation of gene expression by recognizing specific promoter elements and initiating transcription. RpoN-dependent promoters do not have conserved -35 and -10 elements typically found in the promoters recognized by sigma factors in the σ^70 ^family. Instead, a GG dinucleotide around position -24 and a GC dinucleotide around position -12 with respect to the transcription initiation site are highly conserved among RpoN-dependent promoters [1]. The consensus of 186 RpoN-dependent promoter elements from 47 bacterial species was reported to be mrNrYTGGCACG...4bp...TTGCWNNw [2]. In contrast to σ^70 ^family sigma factors, RpoN is able to bind to a promoter without the core RNA polymerase (RNAP) [1]. RNAP containing RpoN (RNAP/RpoN) can form a stable closed complex with the promoter. In addition, RNAP/RpoN requires a transcription factor, the enhancer-binding protein (EBP), for initiation of transcription. Some of the EBP family members are response regulators in two-component regulatory systems [3]. RpoN was first identified in *Escherichia coli *and was reported to regulate the transcription initiation of nitrogen assimilation genes [4]. Since then, RpoN homologs have been identified in bacteria from different phylogenetic origins and are involved in regulation of genes related to a diverse functional categories [5], including genes for pili and flagella biosynthesis and quorum sensing in *Pseudomonas aeruginosa *[6-8], C_4_-dicarboxylate transport in *Mesorhizobium ciceri *[9] and carbon metabolism in Gram positive bacteria, *Bacillus subtilis *and *Listeria monocytogenes *[10-13].

*Geobacter *species are important agents in the bioremediation of subsurface environments contaminated with organic or metal contaminants [14]. They also appear to be the primary contributors to current production in microbial fuel cells harvesting electricity from the environment [15]. Some physiological responses of *Geobacter *species can be predicted with constraint-based genome-scale metabolic models that determine the optimal flux of metabolites for a given environmental condition [16]. The ability to predictively model the physiological responses of environmentally relevant microorganisms to a wide diversity of environmental conditions is a major goal of environmental biotechnology [14,17]. However, the current version of these models lacks regulatory modules that could increase their predictive value. Progress has been made using a combination of bioinformatic tools and molecular biological methods to identify regulatory components, such as operon structures, promoter elements, and transcription factors and their binding sites, as well as global transcriptomic and proteomic expression patterns that provide the basis for building regulatory modules in *G. sulfurreducens *[18-21]. Sigma factors are key to constructing bacterial transcriptional regulatory networks. In *G. sulfurreducen*s, homologs of RpoD, RpoS, RpoH, RpoE and FliA of the σ^70 ^family have been identified and physiological roles of *G. sulfurreducens *RpoS [22,23], RpoH [24] and RpoE (G. Reguera et al, unpublished) have been elucidated.

An ortholog of the *rpoN *gene (GSU1887) is present in the *G. sulfurreducens *genome [19]. It encodes a protein, which shares a considerable degree of similarity to RpoN sigma factors from other bacteria. We report here that an *rpoN *deletion mutant could not be isolated under conditions tested in this study. In order to identify the components of the RpoN regulon in *G. sulfurreducens*, genome-wide microarray transcriptional profiling of an RpoN over-expression strain and genome-wide prediction of RpoN-regulated promoters were employed. We discuss below our findings that RpoN-dependent genes carry out important functions that may contribute to the reasons why no viable *rpoN *deletion mutants could be obtained.

## Results

### The *Geobacter sulfurreducens rpoN *gene cluster

An *rpoN *ortholog (GSU1887), which encodes the RpoN sigma factor, is present in the *G. sulfurreducens *genome. Phylogenetic analysis showed that *G. sulfurreducens *RpoN is very similar to other experimentally characterized RpoN sigma factors, such as the *Escherichia coli *RpoN [19]. The *G. sulfurreducens *RpoN polypeptide displays characteristic structures of the members of the RpoN family, including an N-terminal glutamine-rich region (the first 50 amino acids), a C-terminal X-link, a helix-turn-helix (HTH) DNA-binding motif, and an RpoN box (ARRTVTKYRE) [25].

**Figure 1:**
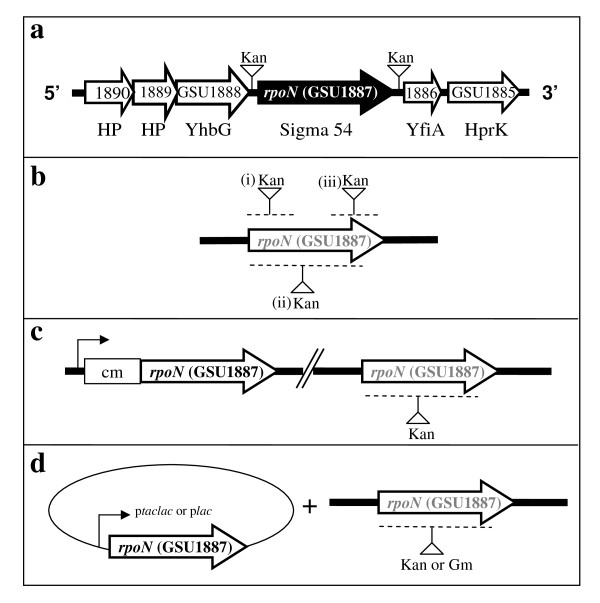
**The *rpoN *gene cluster and the mutation schemes**. (a) Genes surrounding *rpoN *are shown as open arrows. HP: conserved hypothetical protein with unknown function; YhbG: ABC transporter, ATP binding protein; YfiA: ribosomal subunit interface-associated sigma-54 modulation protein; HprK: Hpr(Ser) kinase/phosphorylase. Insertion of a kanamycin resistance cassette upstream or downstream of the intergenic region of the *rpoN *gene resulted in viable mutants (a). (b) Scheme showing attempts of construction of deletion of (i) the 5'-end, (ii) the whole, or (iii) the 3'-end of the *rpoN *coding region. (c) An extra copy of the *rpoN *gene was inserted on the chromosome and was under the control of the chloramphenicol resistance cassette promoter. (d) An extra copy of the *rpoN *gene was introduced *in trans *under the control of a *lac *promoter (constitutively expressed) or a *taclac *promoter (IPTG-inducible). The position of insertion of the antibiotic resistance cassette (kanamycin, Kan or gentamycin, Gm) is indicated with an inverted triangle and a vertical bar. The regions which were attempted to replace with the antibiotic resistance cassette insertion are indicated by dashed line.

Analysis of the chromosomal region surrounding the *G. sulfurreducens rpoN *gene revealed that its downstream genes encode a homolog of a ribosomal subunit interface protein (GSU1886) whose N-terminal domain is homologous to the RpoN modulation protein found in *Klebsiella pneumoniae *[26] and a homolog of Hpr (Ser) kinase/phosphorylase (GSU1885). The upstream genes encode an ATP binding protein (GSU1888) and two conserved proteins (GSU1889 and GSU1890) with unknown function (Figure 1a).

In order to understand the physiological role of RpoN and of genes it controls in *G. sulfurreducens*, construction of a deletion mutant of the *rpoN *gene was attempted. However, an *rpoN *mutant could not be isolated under different growth conditions using media with different electron acceptors (fumarate or Fe(III) citrate) and amendments (glutamine or glutamate) (Table 1). Different mutagenesis strategies were attempted, including deletion of the whole coding region of the *rpoN *gene, only the 5'-end of the *rpoN *coding region, or only the 3'-end of the *rpoN *coding region (Figure 1b). No viable mutants were derived from above attempts. We also constructed an *rpoN *diploid strain (DLCN43) in which an extra copy of the *rpoN *gene was integrated into the chromosome and controlled by the promoter from a chloramphenicol resistance cassette (Figure 1c). Attempts of deleting and replacing the original *rpoN *gene from DLCN43 also failed. Furthermore, an extra copy of the *rpoN *gene under the control of a constitutively active *lac *promoter, or under the control of an IPTG (isopropyl-β-D-thiogalactoside)-inducible *taclac *promoter was introduced to the wild type strain *in trans *(Figure 1d). Attempts of deleting the chromosomal *rpoN *gene yielded viable isolates. However, further analysis of these isolates indicated that none of them had deletion of the chromosomal *rpoN *gene. In contrast, insertion of a kanamycin resistance cassette at the intergenic regions immediately upstream or downstream of the *rpoN *gene yielded viable mutants, suggesting that the inability to isolate an *rpoN*-deletion mutant was not due to the polar effects of the kanamycin resistance cassette. The conditions and mutagenesis methods used in this work for our attempts to isolate a null *rpoN *mutant are summarized in Table 1, and mutation scheme is illustrated in Figure 1.

**Table 1:** List of mutagenesis and selection media for attempts to generate a null *rpoN *mutant.

Mutagenesis	Selection medium (electron donor/acceptor)
1. Deletion and replacement of the *rpoN *gene by double-crossover (Figure 1b)	Acetate/fumarate
	Acetate/Fe(III) citrate
	Acetate/Fe(III) citrate amended with glutamine
	Acetate/Fe(III) citrate amended with glutamate
	Acetate/Fe(III) citrate amended with both glutamine and glutamate

2. Deletion and replacement of the 5'-end of the *rpoN *gene by double-crossover (Figure 1b)	Acetate/fumarate
	Acetate/Fe(III) citrate

3. Deletion and replacement of the 3'-end of the *rpoN *gene by double-crossover (Figure 1b)	Acetate/fumarate
	Acetate/Fe(III) citrate

4. Deletion and replacement of the upstream intergenic region of the *rpoN *gene by double-crossover (Figure 1a)	Acetate/fumarate

5. Deletion and replacement of the downstream intergenic region of the *rpoN *gene by double-crossover (Figure 1a)	Acetate/fumarate

6. Integration of a linear DNA fragment on the chromosome, providing another copy of the *rpoN *gene on another location of the chromosome (Figure 1c)	Acetate/fumarate
	Acetate/Fe(III) citrate

7. Integration of a linear DNA fragment on the chromosome, providing another copy of the *rpoN *gene *in trans*, which is either constitutively expressed by a *lac *promoter, or is IPTG inducible (Figure 1d)	Acetate/fumarate
	Acetate/Fe(III) citrate
	Acetate/Fe(III) citrate amended with both glutamine and glutamate

### RpoN expression patterns and over-expression of RpoN

**Figure 2:**
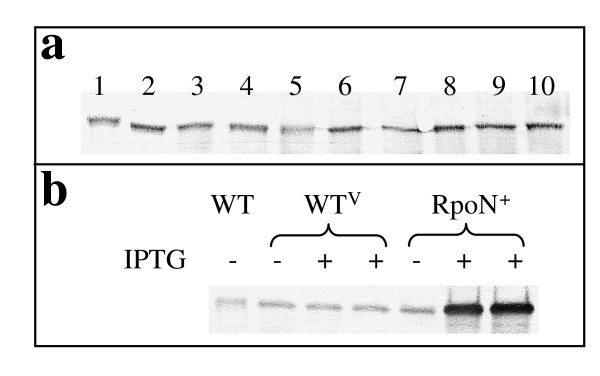
**RpoN expression**. (a) RpoN expression under different growth conditions. 1: NBAF; 2: NBH_2_F; 3: NBLF; 4: ammonium-free NBAF; 5: FWAFC; 6: FWH_2_FC; 7: FWLFC; 8: FWAF; 9: FWH_2_F; 10: FWLF. Media abbreviations were detailed in Methods. (b) RpoN over-expression. Total protein (5 μg) was separated by 10% SDS-PAGE and analyzed by Western blot analysis with the RpoN-specific antiserum. Two biological samples were shown for IPTG-induced WT^V ^and RpoN^+ ^strains. IPTG was added at final concentration 1 mM.

In order to understand RpoN expression under different growth conditions, Western blot analysis was performed using anti-sera against RpoN with cell extracts prepared from cultures grown with a variety of electron donors/acceptors. RpoN levels were similar in cultures grown in the presence of ammonium or when nitrogen fixation was required in ammonia-free medium, and RpoN was constitutively expressed under all other conditions tested (Figure 2a).

Because an *rpoN *deletion mutant could not be obtained, a strain in which the *rpoN *gene was over-expressed under the control of the IPTG-inducible *taclac *promoter was generated to gain insights into the function of RpoN. The strain capable of over-expressing RpoN (DL1/pCDrpoN) and the control strain harboring the empty vector pCD341 (DL1/pCD341) were designated RpoN^+ ^and WT^V^, respectively, for simplification. The over-expression of the RpoN protein in RpoN^+ ^after induction was confirmed using Western blot analysis (Figure 2b). The abundance of the RpoN protein in the RpoN^+ ^strain was 5.4 times more than that of the wild type with IPTG induction, but it stayed at a similar level as that of the wild type in the absence of IPTG. The WT^V ^strain, with or without the addition of IPTG, had levels of RpoN similar to those in the wild type strain.

**Figure 3:**
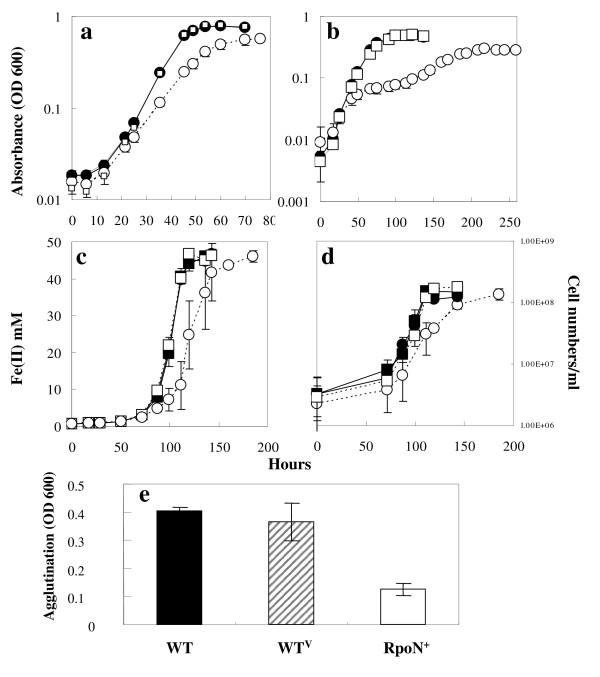
**Characterization of the RpoN over-expression strain**. Cell growth with fumarate as an electron acceptor was monitored by absorbance at 600 nm (a)(b). (a) acetate as the electron donor and fumarate as the electron acceptor (NBAF medium); (b): ammonia-free NBAF. Growth with Fe(III) as an electron acceptor was monitored by Fe(II) production (c) as well as cell numbers (d). Filled square: the WT^V ^strain without IPTG; Empty square: the WT^V ^strain with IPTG. Filled circle: the RpoN^+ ^strain without IPTG; empty circle: the RpoN^+ ^strain with IPTG. (a)-(d): Data are means ± standard deviations of triplicates. The production of pili was measured by agglutination assays (e). Data are means ± standard deviation of triplicates from two independent experiments (e).

When induced with IPTG, the RpoN^+ ^strain grew slower and had a longer lag phase than the WT^V ^strain with either fumarate or Fe(III) citrate as the electron acceptor and acetate as the electron donor (Figure 3a, c &3d). The effect of over-expressing RpoN on growth was more pronounced when cells were grown in media lacking ammonia (Figure 3b). The doubling times for the WT^V ^and the RpoN^+ ^strains were 7.5 and 9.7 hours, respectively, in the presence of IPTG in the NBAF medium (Figure 3a). Under nitrogen fixation conditions, the doubling times for the WT^V ^and the RpoN^+ ^strains were 12.5 and 51.3 hours, respectively, in the presence of IPTG in the ammonium-free NBAF medium (Figure 3b). For FWAFC media, the doubling times were 9.6 and 14.9 hours with IPTG for WT^V ^and RpoN^+^, respectively (Figure 3d). The RpoN^+ ^strain grew similarly with the WT^V ^strain in the absence of IPTG in all media. These results suggest that over-expression of RpoN inhibited growth under various conditions.

### The RpoN regulon identified by genome-scale transcriptome analysis and prediction of RpoN-dependent promoters

To elucidate the function of RpoN in *Geobacter *species, the transcriptome of the RpoN^+ ^and the WT^V ^strains was compared in order to identify those genes whose transcription is regulated by RpoN. Due to different growth rates between the two strains in the NBAF medium (Figure 3), total RNA was isolated independently from three sets of WT^V ^and RpoN^+ ^cultures during the exponential growth phase, about OD600 = 0.3–0.35 and 0.2–0.25 for the WT^V ^and RpoN^+ ^strains, respectively.

With a 1.5 fold-change cutoff and a relatively strong cutoff for false discovery rate (FDR) of p < 0.0005, the RpoN^+ ^strain was found to have 138 genes with increased transcript levels and 59 genes with decreased transcript levels when compared to the WT^V ^strain. The fold change of the transcription level of the *rpoN *gene itself was 6.61 higher in the RpoN^+ ^strain than in the WT^V ^strain, which is in agreement with protein over-expression results obtained by the Western blot analysis. A complete list of the differentially expressed genes and their expression ratio is provided as additional files (see Additional files 1 &2). The 196 genes with significant changes in expression level in the RpoN^+ ^strain were assigned to 16 functional groups. Most of them encoded hypothetical proteins or proteins with unknown function (63 genes). The top three categories of genes with annotated functions consisted of genes associated with protein synthesis (33), energy metabolism (21 genes), and transport and binding (12).

In addition to observing the RpoN regulon by using gene expression microarrays, we employed computational analysis using the PromScan software [27] to identify RpoN recognition sequence elements within the *G. sulfurreducens *genome. The search identified 798 putative RpoN recognition sequence elements with scores ≥ 80 (data not shown). Among these sequence elements, 467 elements were located upstream of protein-coding genes, in the same strand orientation as their potential target genes. Of these possible promoter elements that could potentially regulate transcription of protein-coding genes, 110 were located in noncoding regions. We considered these 110 sequence elements to be the most likely RpoN-regulated promoters. Their sequences and genome locations are provided in Additional file 3, while their predicted target genes and operons are provided in Additional file 4. The consensus sequence of these 110 predicted *G. sulfurreducens *RpoN-regulated promoter elements listed in Additional file 3 was TT**GG**CACGGTTTTT**GC**T, where the -24 GG and the -12 GC dinucleotides are in bold. The highest scoring RpoN-regulated promoter was located upstream of the flagella biosynthesis operon containing the *fliA *gene encoding the RNA polymerase sigma factor FliA (σ^28^) (see Additional file 4).

Genome locations of the 110 RpoN-dependent sequence elements identified using the PromScan analysis were cross-examined with the list of genes identified by the transcriptome analysis with a fold change cutoff of 1.25. The results of this comparison are listed in Additional file 5, and selected operons encoding genes related to nitrogen assimilation, appendages and solute transport are listed in Table 2. These combined data showed that RpoN regulatory elements can be found in not only up-regulated genes, but also down-regulated genes. The increased transcript levels observed for some genes could result from increased RpoN availability for transcription initiation that is the limiting factor at a normal *rpoN *transcript level. The decreased transcript levels for some genes could be due to the fact that RpoN alone can bind to the -24/-12 elements without the core RNAP, and therefore the promoter regions may not be accessible by other sigma factors, or less core RNAP may be available in the excess of RpoN [1,28].

**Table 2:** Genes containing RpoN-dependent promoters identified by the PromScan analysis and the transcriptome analysis.

Operons^§^	Genes	Annotations	Fold changes*	PromScan score
**Amino acid biosynthesis and Nitrogen assimilation**				

Glutamine synthase (GS)	GSU1835 (*glnA*)^†^	glutamine synthetase (GS)	+1.39	80
	GSU1836 (*glnB*)^†^	nitrogen regulatory protein PII	+1.77	

Glutamate synthase (GOGAT)	GSU1235	hypothetical protein	N. D.	88
	GSU1236	hypothetical protein	N. D.	
	GSU1237	pyridine nucleotide-disulphide oxidoreductase family protein	-1.44	
	GSU1238	iron-sulfur cluster-binding protein	N. D.	
	GSU1239 (*gltB*)^†^	glutamate synthase-related protein (GOGAT)	N. D.	

Nitrogen assimilation	GSU2802	NAD(+) – dinitrogen-reductase ADP-D-ribosyltransferase	N. D.	86
	GSU2803	dinitrogenase iron-molybdenum cofactor family protein	-1.21	
	GSU2804	ferredoxin family protein	-1.24	
	GSU2805 (*nifX*)	nitrogenase molybdenum-iron cofactor biosynthesis protein NifX	-1.27	
	GSU2806 (*nifEN*)^†^	nitrogenase molybdenum-iron cofactor biosynthesis protein NifEN	-1.34	

**Appendages and Motility**				

Flagella biogenesis	GSU3050 (*flgA*)	flagella basal body P-ring formation protein FlgA	-1.34	93
	GSU3051 (*flgG*)	flagellar basal-body rod protein FlgG	N. D.	
	GSU3052 (*flgG*)	flagellar basal-body rod protein FlgG	-1.19	
	GSU3053 (*fliA*)^†^	RNA polymerase sigma factor for flagellar Operon/gene/gene	-1.50	
	GSU3054	ParA family protein	-1.21	
	GSU3055 (*flhF*)	flagellar biosynthetic protein FlhF	-1.34	
	GSU3056 (*flhA*)	flagellar biosynthetic protein FlhA	-1.42	

Flagella basal body	GSU0407 (*flgB*)	Flagellar basal-body rod protein FlgB	-1.38	92
	GSU0408 (*flgC*)	Flagellar basal-body rod protein FlgC	-1.31	

Flagella biogenesis	GSU0420 (*fliL*)^†^	flagellar protein FliL	-1.47	86
	GSU0421 (*fliM*)	flagellar motor switch protein FliM	-1.35	
	GSU0422 (*fliN*)	flagellar motor switch protein FliN	-1.93	
	GSU0423 (*fliP*)	flagellar biosynthetic protein FliP	N. D.	
	GSU0424 (*fliQ*)	flagellar biosynthetic protein FliQ	-1.32	
	GSU0425 (*fliR*)	flagellar biosynthesis protein FliR	-1.72	
	GSU0426 (*flhB*)	flagellar biosynthetic protein FlhB	-1.11	

Flagella biogenesis	GSU3040	hypothetical protein	N. D.	86
	GSU3041	carbon storage regulator	-1.04	
	GSU3042 (*flgL*)	flagellar hook-associated protein FlgL	-1.05	
	GSU3043 (*flgK*)	flagellar hook-associated protein FlgK	-1.05	
	GSU3044	hypothetical protein	-1.21	
	GSU3045 (*flgM*)	negative regulator of flagellin synthesis FlgM	-1.13	
	GSU3046 (*flgJ*)^†^	flagellar protein FlgJ-like protein	-1.41	

**Metabolisms**				

Formate dehydrogenase	GSU0777(*fdnG*)^†^	formate dehydrogenase, major subunit, selenocysteine-containing	N. D.	90
	GSU0778	formate dehydrogenase, iron-sulfur subunit	N. D.	
	GSU0779	formate dehydrogenase, *b*-type cytochrome subunit	-1.19	
	GSU0780	formate dehydrogenase accessory protein FdhD	N. D.	
	GSU0781	twin-arginine translocation protein, TatA/E family	-1.41	

**Solute transporter**				

Fumarate/succinate exchanger	GSU2750	hypothetical protein	+1.30	83
	GSU2751 (*dcuB*)^†^	C_4_-dicarboxylate transporter (DcuB)	+1.63	

§Operon predictions in *G. sulfurreducens *are described in [18].

^†^Genes were discussed in the text and/or RpoN regulatory elements have been confirmed in their promoter regions by primer extension analyses

*Not all genes listed in the table matched the criteria for ≥ 1.25 fold-change cutoffs by the gene expression microarray analysis as described in Methods, but these genes are listed because one or more genes from the same operon matched the cutoff criteria.

N.D.: Not detected.

Both microarray transcriptome analysis and computational prediction of RpoN promoter elements concluded that the *dcuB *gene, which encodes the fumarate/succinate exchanger (C_4_-dicarboxylate transporter), is RpoN-dependent. Both analyses also indicated that genes encoding components for nitrogen assimilation, such as glutamine synthetase (GS) (GSU1835), are RpoN-dependent (Table 2). Furthermore, our computational analysis identified an RpoN promoter upstream of the glutamate synthetase (GSU1239, GOGAT) operon. The microarray analysis indicated that the gene encoding glutamate dehydrogenase (GDH) (GSU1305) is RpoN-dependent.

*In silico *modeling analysis was utilized to understand the role of GDH, GS and GOGAT enzymes in *G. sulfurreducens *metabolism. The growth of *G. sulfurreducens *was simulated under different growth conditions, varying electron donors, electron acceptors, and nitrogen sources. *In silico *modeling analysis suggested that missing both GS and GOGAT enzymes or GDH, GS and GOGAT is lethal under all growth conditions.

Other genes identified by both the transcriptome analysis and the computational promoter prediction include those encoding flagella biosynthesis, formate dehydrogenase, alcohol dehydrogenase, and acetyl-CoA carboxylase (Table 2 and Additional file 5).

An RpoN-dependent regulatory element, located upstream of the *pilA *gene encoding pilin, the building block for nanowires [29], was identified by the PromScan software in this study and by 5'-RACE analysis in an earlier study [30]. Therefore, the effect of RpoN-overexpression was tested on the pili formation via a cell agglutination assay at 25°C [31]. In the presence of IPTG, the RpoN^+ ^strain displayed less agglutination at 25°C than WT^V ^and WT (Figure 3e), which strongly suggests that RpoN is involved in pili biogenesis.

Genes that were differentially expressed in the RpoN^+ ^strain but for which no RpoN-dependent regulatory elements could be found include those encoding components of stress response/molecular chaperones, central metabolism, extracellular electron transfer, and genes encoding regulatory proteins (see Additional files 1 &2), suggesting their possible indirect regulation by RpoN or a possibility that their RpoN promoters may be too divergent from promoters in other bacteria to be detected using computational approaches. Alternatively, the physiological states caused by the overexpression of RpoN in the RpoN^+ ^strain, which were different from those in the WT^V ^strain, such as slower growth, might affect gene regulation.

### Evaluation of predicted RpoN-dependent promoters via primer extension analysis

**Figure 4:**
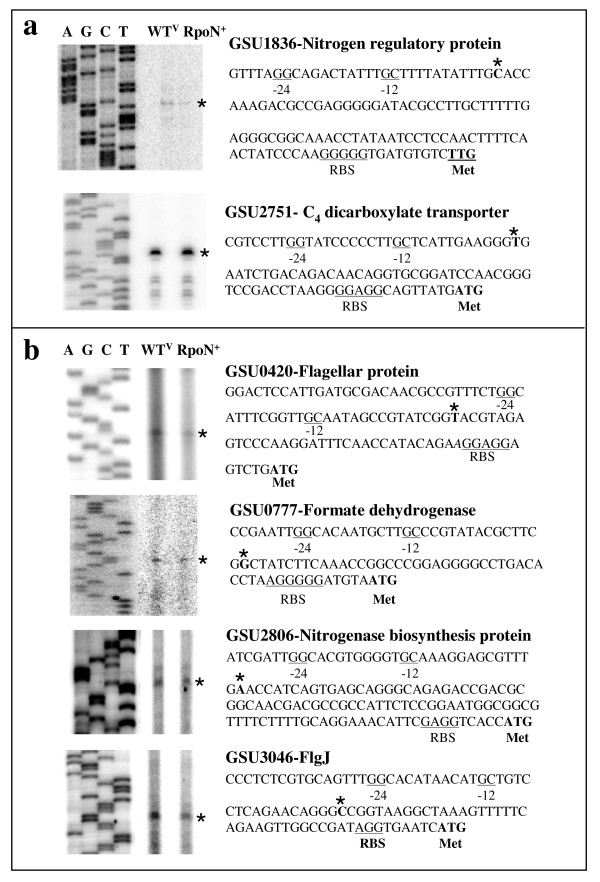
**RpoN-dependent gene expression**. Representative genes, (a) GSU1836 and GSU2751 (up-regulated in the RpoN^+ ^strain), and (b) GSU0420, GSU0777, GSU2806 and GSU3046 (down-regulated in the RpoN^+ ^strain) identified by the microarray analysis were further analyzed by primer extension assays. The results of the primer extension assays and their promoter regions are shown. The 5' ends of mRNA are indicated by asterisks. The putative -24/-12 elements and RBS are underlined. Translation start codons are shown in bold and are indicated by Met.

In order to further validate the results of the microarray transcriptome analysis and of the computational promoter prediction and to examine promoters regulating differentially expressed genes, primer extension assays were carried out on 12 selected operons or singleton genes, including seven genes with increased expression and five genes with decreased expression in the microarray analysis (Table 3). These selections included 1) genes involved in cellular functions that were reported in other bacteria to be RpoN-dependent, such as flagella biosynthesis and nitrogen assimilation; or 2) genes involved in physiological functions that were not previously reported to be RpoN-dependent in other bacteria, but were predicted to have conserved RpoN-dependent -24/-12 promoter elements in their regulatory regions, or 3) genes encoding enzymes that are essential for cell growth. The trend of changes in transcript levels observed in the primer extension assays was similar to that reported in the microarray analysis (Figure 4, Table 3 and Additional file 6), with an exception of GSU1836 (*glnB*), which encodes a PII nitrogen regulatory protein.

**Table 3:** Summary of 12 genes whose 5' ends of mRNA were analyzed by primer extension assays.

Gene #	ID	Promoter elements validated	In agreement with microarray data	In agreement with PromScan
Up-regulated according to microarray analysis				

GSU0364	*ppcB*	RpoD	Yes	Yes
GSU1836	*glnB*	**RpoN**	No	Yes
GSU2005	ABC transporter	RpoD	Yes	No
GSU2302	Trehalose phosphatase	RpoD	Yes	Yes
GSU2490	Oxalate-formate antiporter	RpoD	Yes	No
GSU2751	*dcuB*	**RpoN**	Yes	Yes
GSU3206	*dksA*	RpoD	Yes	No
				
Down-regulated according to microarray analysis				

GSU0420	*fliL*	**RpoN**	Yes	Yes
GSU0777	*fdnG*	**RpoN**	Yes	Yes
GSU0939	*PII*	RpoD	Yes	No
GSU2806	*nifEN*	**RpoN**	Yes	Yes
GSU3046	*flgJ*	**RpoN**	Yes	Yes

**Figure 5:**
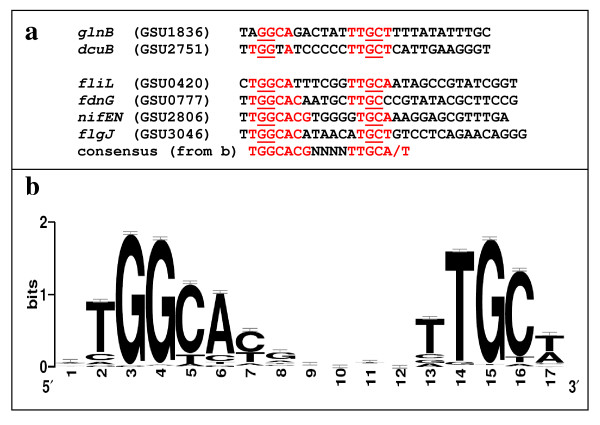
***G. sulfurreducens *RpoN-dependent promoter elements**. (a) Alignment of *G. sulfurreducens *RpoN-dependent promoters identified by primer extension assays in this study. Conserved nucleotides which are the same to the consensus sequences from (b) are labeled in red. (b) Sequence logo of 110 *G. sulfurreducens *RpoN-regulated promoters predicted by PromScan in non-coding regions upstream of target protein-coding genes.

Eight genes out of the 12 selected genes had promoter elements identified by the primer extension assays to match those identified by the computational predictions, and six of these eight genes were found to be regulated by promoter sequences highly similar to other bacterial RpoN promoter elements (Table 3). These six RpoN-regulated genes were GSU0420 (*fliL*), GSU1836 (*glnB*), GSU2806 (*nifEN*), GSU3046 (*flgJ*), GSU2751 (*dcuB*) and GSU0777 (*fdnG*) (Figure 4 &5 and Table 3). Alignment of these six *G. sulfurreducens *RpoN promoter elements and of the promoter element upstream of the *pilA *gene identified in the previous study [30] was shown (Figure 5).

While conserved RpoN-regulated promoters were predicted for other four genes/operons (GSU0939, GSU2005, GSU2490 and GSU3206) by the computational tools (see Additional file 4), no -24/-12 RpoN-dependent promoter elements could be identified by primer extension assays for these genes/operons (see Additional file 6). It is possible that the predicted RpoN-regulated promoter elements for these four genes/operons are activated only under certain conditions, such as nitrogen-fixing conditions, which were different from the conditions for the primer extension assays.

In conclusion, the primer extension analyses showed that RpoN-regulated promoters are located upstream of genes for a fumarate/succinate exchanger (GSU2751, *dcuB*), glutamine synthetase (GSU1836-1835 operon, *glnB-glnA*), flagella biosynthesis proteins (GSU0420–0426, and GSU3040–3046), nitrogen assimilation enzymes (GSU2802–2806), and a formate dehydrogenase (GSU0777–0781). In addition, an RpoN-regulated promoter was also identified for the *pilA *gene (GSU1496) by 5'-RACE analysis [30].

## Discussion

### The *G. sulfurreducens *RpoN regulon

The *G. sulfurreducens *RpoN regulon was identified using the microarray transcriptome analysis combined with the computational analysis. Both methods indicated that RpoN plays an important role in influencing the expression of a number of genes that are important for growth in subsurface environment and in microbial fuel cells. Most evidently, both methods demonstrated that the *G. sulfurreducens *RpoN controls the expression of a fumarate/succinate exchanger (DcuB), which is essential under fumarate respiration [32]. Thus, no viable *rpoN *deletion mutant was isolated when fumarate was the terminal electron acceptor. This is the first report to our knowledge identifying a -24/-12 RpoN-dependent promoter element in the *dcuB *regulatory region. In other bacteria, such as *E. coli*, the *dcuB *gene is transcribed by RNA polymerase in complex with the RpoD sigma factor, and its expression is under a hierarchical control involving FNR, CRP, and a two-component regulatory system [33].

Glutamate and glutamine, the essential biomass components and major intracellular nitrogen donors, are the products of ammonia assimilation and are synthesized in bacteria via two pathways [34]. The *G. sulfurreducens *RpoN controls the expression of enzymes involved in both pathways for ammonia assimilation, namely, 1) the GDH-dependent pathway, in which glutamate is synthesized by reductive amination of 2-ketoglutarate and 2) the GS-GOGAT pathway, in which GS converts glutamate and ammonia to glutamine and GOGAT transfers the amide group from glutamine to 2-ketoglutarate. Therefore, deleting the *rpoN *gene would result in deficiencies in ammonia assimilation and thus, cell death. In *E. coli*, the GS-GOGAT pathway is used under energy-rich and nitrogen-limiting conditions and the expression of both enzymes is under the control of RpoN, whereas the GDH pathway is employed under energy-limiting and excess ammonium conditions, and the transcription of GDH is controlled by RNAP/RpoD [34-36]. In fact, the expression of other known bacterial GDHs is controlled by RNAP/RpoD, including those in *Neisseria meningitides, Klebsiella aerogenes, Psychrobacter sp, Streptococcus pneumoniae and Pseudomonas aeruginosa *[37-42].

Acetate is a primary electron donor for *Geobacter *species in soils and sediments [43], especially during groundwater uranium bioremediation [44]. The microarray study suggests that RpoN positively regulates the expression of genes essential for acetate oxidation coupled to metal reduction such as fumarase (GSU0994) and acetyl-CoA transferases (GSU0174 and GSU0490) [45]. The inability to isolate an *rpoN *null mutant could also be due to the poor expression of these essential TCA cycle enzymes in the absence of RpoN. The ability to recover such a mutant with Fe(III) as the electron acceptor would also be limited due to low expression of the cytochromes essential for Fe(III) reduction, such as the *omcB *gene [46], which was up-regulated in the RpoN^+ ^strain (see Additional file 1).

Flagella are considered to play an important role in Fe(III) oxide reduction by *Geobacter *species [47]. The regulatory regions from both operons containing GSU0420 and GSU3046 encoding flagella proteins contain sequences homologous to RpoN-dependent promoter sequences (Figure 4 and 5). As presented in Table 2 and Additional file 4, the operon containing the *fliA *gene (σ^28^, GSU3053) along with other flagella biosynthesis genes (*flg-1*, *flg-2*, *flh-A*, and others) was predicted to have the highest scoring RpoN-regulated promoter, indicating that this promoter was highly conserved and that σ^28 ^gene expression in *G. sulfurreducens *is likely regulated by RpoN. Interestingly, these results are similar to *Campylobacter *spp and *Vibrio *spp, in which RpoN regulates the σ^28 ^gene expression (see reviews and references within [48-50]). In other bacterial species, e.g. *Salmonella enterica *serovar *Typhimurium*, the regulation of *fliA *is controlled by the FlhCD transcriptional regulator [50]. FlhCD is absent from the *G. sulfurreducens *genome, which also appears to lack FlhCD binding sites [19]. It is interesting to note that *C. jejuni *and *V. cholerae *use pili and flagella to achieve virulence [50], whereas in *Geobacter *species, flagella and pili are implicated in extracellular Fe(III) reduction [29,47] and higher power production in microbial fuel cells [51].

### Unique features of the *G. sulfurreducens *RpoN sigma factor

In most bacteria that have been studied, the *rpoN *gene deletion resulted in viable mutants or mutants requiring certain nutrient addendum [8,52,53]. However, despite the high amino acid sequence similarity of the *G. sulfurreducens *RpoN to other bacterial homologs, an *rpoN *null mutant was not obtained after multiple attempts. The only other case in which the *rpoN *gene is essential was reported in *Myxococcus xanthus*, another *delta*-*proteobacterium*, for reasons that have yet to be elucidated [54].

This work also suggested that the expression of RpoN in *G. sulfurreducens *is under a tight control in a complex manner for several reasons: (1) over-expression of RpoN inhibited growth under various growth conditions, (2) over-expression of RpoN induced up-regulation of genes involved in stress responses (see Additional file 1), and (3) our inability to isolate a viable *rpoN *deletion mutant even in the presence of another copy of the *rpoN *gene, *in trans *or *in cis*, which was under the control of artificial promoters such as the *lac *promoter or the promoter from the chloramphenicol resistance gene. Furthermore, the *G. sulfurreducens *genome contains 28 genes encoding transcription factors from the EBP family, whose members are required for transcription initiation directed by RNAP/RpoN. This number of the EBPs is much higher than that found in most bacteria. For instance, *E. coli*, which has a larger genome (4.6 Mbp) than *G. sulfurreducens *(3.8 Mbp), has only 12 EBPs [4,55]. Approximately half of the EBPs in *G. sulfurreducens *belong to two-component regulatory systems. This further suggests that cellular responses to various environmental conditions are directed by gene expression regulated by RpoN. It has been reported for other bacteria that EBP-encoding genes are often located adjacent or close to their target promoters ([3] and references within). This was also found to be the case for some of the EBPs in *G. sulfurreducens*. It has been shown that PilR, a member of the EBP family, regulates the *pilA *gene, which has an RpoN-dependent promoter and is located immediately downstream of the *pilR *gene [30]. Genes encoding EBPs are also located upstream of the *dcuB *and *fdnG *genes, which were shown to contain an RpoN-dependent promoter (Figure 4). In addition, a gene encoding an EBP is located upstream of GSU3364, which was predicted to contain an RpoN-dependent promoter (see Additional file 4). However, it appears that *G. sulfurreducens *EBPs are not always located adjacent or close to their target promoters for the cases of *fliL*, *glnB *(GSU1836), *nifEN*, and *flgJ*, which were shown to contain an RpoN-dependent promoter in their regulatory regions (Figure 4).

### Data analysis-combining computational prediction with microarray analysis

Due to the absence of an RpoN deletion mutant, our analyses were restricted to the use of an RpoN over-expressing strain. Activation of RpoN-dependent transcription requires the presence of EBPs activated by modification such as phosphorylation, and therefore, an increase in the amount of RpoN alone would not directly lead to increasing expression of every RpoN-regulated gene. This is exactly what we have observed with our microarray analysis. With a relatively strong cutoff for false discovery rate of p < 0.0005, the majority (~85%) of differentially expressed genes had fold changes between 1.50 and 2. When the results from the computational prediction are compared with the results from the transcriptome analysis, the use of an arbitrary cutoff level may not be able to detect all RpoN-regulated genes in the RpoN over-expressing strain. For instance, from the transcriptome analysis, the fold change of the *nifEN *gene encoding a subunit of nitrogenase complex was -1.34 (Table 2), while an RpoN-dependent promoter for this gene was identified in *G. sulfurreducens *(Figure 4) and in other species [34]. Therefore, it is likely that the fold change in expression of the *nifEN *gene and other genes did not reach the 1.5 threshold due to complexity of gene regulation in the RpoN over-expressing strain. In order to provide a broader list of potential RpoN targets, we therefore provided a list of genes predicted to be under control of RpoN-regulated promoters (see Additional file 4), with predicted sequences. To increase the possibility of capturing even weaker effects of RpoN on gene expression, the threshold level for expression changes was set at 1.25 for comparison of the microarray transcriptome analysis and the computer prediction (Table 2 and Additional file 5). By lowering the cutoff of fold changes and by using additional validation by the primer extension assays, we were able to confirm RpoN regulatory elements located upstream of operons/genes identified by both methods (Table 2 &3), suggesting that this strategy is feasible.

The results from the transcriptome analysis and the primer extension assays showed that RpoN-dependent promoters can be found upstream of both up- and down-regulated genes in the RpoN^+ ^strain. For example, increased transcript levels observed for some genes, such as *dcuB*, could result from increased RpoN availability for transcription initiation, which is the limiting factor at a normal *rpoN *transcript level. Therefore, such genes were up-regulated with the increase in the RpoN level. In contrast, excess RpoN may inhibit transcription due to lack of proportional increase in EBPs and/or core RNAP, thereby making excess RpoN an inhibitor or repressor instead of an activator. This might be explained by the degree of the conservation of the RpoN-recognition sequences. When the RpoN-recognition sequences of *glnB, dcuB, fliL, fdnG, nifEN*, and *flgJ*, for which their RpoN-recognition sequences were identified by the primer extension assays (Figure 4), were analyzed, it was found that the RpoN-recognition sequences of the genes repressed in the overexpressing strain (*fliL, fdnG, nifEN*, and *flgJ*) are slightly more similar to the consensus of the RpoN-recognition sequences (10 or 11 nucleotides conserved out of 13 consensus nucleotides) than those for the up-regulated genes (*glnB *and *dcuB*, 9 nucleotides conserved out of 13 consensus nucleotides) (Figure 5a). This slight difference could make significant difference in transcriptional activation and repression. Therefore, RpoN might bind more tightly to the promoters that are more similar to the consensus and inhibit their transcription in the absence of a proportionally increased amount of an EBP for these promoters. Furthermore, the decreased transcript levels for some genes could also be due to "sigma factor antagonism" in which other sigma factor(s) could not access the promoter regions that were engaged by RpoN [28], or because less core RNAP may be available for other sigma factors in the excess of RpoN. It is likely that more than one sigma factor and/or transcriptional regulators can affect gene expression in the same cell. For example, it has been proposed that the transcription of RpoN-dependent promoters is affected by ppGpp and its cofactor DksA through a mechanism in which more core RNAP are available for RNAP/RpoN holoenzyme formation due to the short half life of RNAP/RpoD induced by binding to ppGpp and DksA [56,57]. Alternatively, the physiological states caused by the overexpression of RpoN in the RpoN^+ ^strain, which were different from those in the WT^V ^strain, such as slower growth, and/or growth conditions, which affect gene regulation, such as fumarate respiration, resulted in positive effects on some genes and negative effects on others. For instance, the *dcuB *gene was up-regulated in the RpoN^+ ^strain, because the active EBP was likely present for the activation of the *dcuB *gene under the conditions for the microarray transcriptome analysis, during which cells needed to grow on fumarate, and thus the fumarate/succinate exchanger encoded by the *dcuB *gene was required for growth. In contrast, it is unlikely that the genes for nitrogen fixation such as *nifEN *were essential for growth in the presence of ammonia and thus the active EBP for these genes was scarce, if present, under the conditions for the microarray transcriptome analysis, resulting in the inhibition of these genes in the RpoN^+ ^strain. It is also possible that RpoN may affect regulation of different promoters under a different set of conditions or different stages of cell growth.

## Conclusion

The results presented here demonstrate that *G. sulfurreducens *has an RpoN ortholog, which exhibits typical structural characteristics shared by the RpoN family. However, unlike most of other bacterial *rpoN *genes, the *G. sulfurreducens rpoN *was indispensable for growth under all conditions tested. By combining data from the computational prediction with the microarray analysis of the RpoN over-expression strain, the regulon of *G. sulfurreducens *RpoN was identified, which includes a number of genes that are important for growth in subsurface environments and microbial fuel cells. The *G. sulfurreducens *RpoN regulates the expression of the *dcuB *gene encoding a fumarate/succinate exchanger, which is essential for fumarate respiration. The *G. sulfurreducens *RpoN controls both pathways of glutamate/glutamine syntheses, including the GDH (glutamate dehydrogenase) pathway and the GS/GOGAT (glutamine synthase/glutamate synthase) pathway. Thus, deletion of the *rpoN *gene would hinder cells' ability for ammonia assimilation, and therefore this mutant would not be viable. This study provides information on transcriptional regulatory networks in *G. sulfurreducens*, which would increase the predictive value of the regulatory modules in the genome-wide *in silico *models. Further studies for the RpoN transcriptional network in global regulation in *G. sulfurreducens *are currently underway to fine-tune the regulatory modules in the models.

## Methods

### Bacterial strains and culturing conditions

*Escherichia coli *strain JM109 [*endA-1 recA-1 gyrA-96 thi hsdR-17*(r_k_^-^, m_k_^+^) *relA-1 supE-44 *Δ(*lac-proAB*)(F' *traD-36 proAB lacI*^q^ZΔM15)] [58], or TOP10 [F^-^*mcrA *Δ(*mrr-hsdRMS*-*mcrBC*) Φ 80*lacZΔ*M15 Δ*lacX-74 recA-1 araD-139 *Δ(*ara-leu*)7697 *galU galK rpsL *(Str^R^) *endA*-*1 nupG*] [59] was cultured in LB medium at 37°C with shaking. Targeted gene disruption experiments were performed on *G. sulfurreducens *strain DL1 [60,61] to produce strains DLCN29 (a kanamycin cassette insertion between GSU1888 and 1887 (*rpoN*)), DLCN32 (a kanamycin cassette insertion between GSU1887 and GSU1886) (Figure 1) and an *rpoN *diploid strain DLCN43. *G. sulfurreducens *strains were routinely cultured anaerobically in NB acetate-fumarate (NBAF) or freshwater acetate-Fe(III) citrate (FWAFC) medium at 30°C as previously described [61]. NB and FW are the two basic mineral solutions, and differ mainly in buffering capacity and trace element contents. Acetate (15 mM) and fumarate (40 mM) were the electron donor and electron acceptor, respectively, for the general propagation unless otherwise stated. Both can be substituted with either lactate (20 mM) or hydrogen as an electron donor, or Fe(III) citrate (55 mM) as an electron acceptor when necessary (for a complete media composition please see references [61,62]).

### DNA manipulations

Genomic DNA was extracted with the Qiagen Genomic-tip 100/G. Plasmid DNA and PCR products were purified with the Qiagen mini plasmid purification kits and PCR purification kits, respectively (Qiagen). DNA cloning and other manipulations were carried out according to the methods outlined by Sambrook *et al*. [63]. Restriction enzymes and other DNA-modifying enzymes were from New England Biolabs. Probes for Southern blot analyses were labeled with [α-^32^P]dCTP using the NEBlot kit (New England BioLabs). [α-^32^P]dCTP was from PerkinElmer Life and Analytical Sciences. Qiagen *Taq *DNA polymerase, unless otherwise stated, was used for all PCR amplifications.

### Single-step gene replacement

Sequences were deleted with single-step gene replacement as previously described [64]. To disrupt the intergenic regions either upstream (between *rpoN *(GSU1887) and GSU1888) or downstream (between *rpoN *and GSU1886) of the *rpoN *gene, a linear DNA fragment was generated by recombinant PCR [64,65] from three primary PCR products. For disruption of the intergenic region upstream of the *rpoN *gene, a 2.1 kb linear DNA fragment was composed of three PCR products: (1) the 3' end of GSU1888 (0.5 kb, amplified with primers rpoNU-1 and rpoNU-2); (2) 5' end of the *rpoN *gene (0.5 kb, amplified with primers rpoNU-5 and rpoNU-6); and (3) a kanamycin resistant cassette (Kan^R^) (1.1 kb, amplified with primers rpoNU-3 and rpoNU-4). For disruption of the intergenic region downstream of the *rpoN *gene, three primary PCR reactions were performed to amplify a 2.1 kb linear DNA fragment: (1) the 3' end of *rpoN *[0.5 kb, position to position, amplified with primers rpoND-1 and rpoND-2); (2) 5' end of the GSU1886 gene (0.5 kb, amplified with primers rpoND-5 and rpoND-6); and (3) a Kan^R ^cassette (1.1 kb, amplified with primers rpoND-3 and rpoND-4). Recombinant PCR was performed with these three PCR products as templates with distal primer pairs, rpoNU-1/rpoNU-6 and rpoND-1/rpoND-6 for upstream or downstream intergenic region mutation respectively. PCR conditions were as previously described, except that the annealing temperature was 58°C [64]. All primer sequences used in this work are listed in Additional file 7.

Electroporation, mutant isolation and genotype confirmation were performed as previously described [61,64]. One of each of the mutants, designated DLCN29 and DLCN32, was chosen as the representative strain.

### Construction of an *rpoN *diploid strain of *G. sulfurreducens *(DLCN43)

A 2.5 kb linear DNA fragment containing the chloramphenicol resistance cassette (Cm^R^) followed by the coding region of the *rpoN *gene was constructed using cross-over PCR [65]. The chloramphenicol resistance cassette was amplified with Cm-rpoNF1 (*Cla *I site) and Cm-rpoNR2 using pACYC184 as the template. The *rpoN *gene was amplified with C-rpoNF3 and C-rpoNR4. The two PCR products were joined together by cross-over PCR as described in [64,65]. The resulted PCR product (Cm-rpoN) was Klenow filled-in and ligated to the *Sma *I-cut pLA01 (as described below), resulted in plasmid pLA03.

The plasmid pLA01 is a derivative of pCR2.1-TOPO that the 5'-end of the periplasmic *c*-type cytochrome gene (*ppcA*) which was amplified with primer pair: ppcAF1 and ppcAR2 was cloned into pCR2.1-TOPO using TOPO TA cloning kit (Invitrogen). Therefore, plasmid pLA03 contains the 5'-end *ppcA *followed by Cm-rpoN: the Cm^R ^resistance cassette and the *rpoN *gene, and the 3'-end of *ppcA*. The plasmid pLA03 was linearized and electroporated into *G. sulfurreducens *DL1 and Cm^R ^transformants were selected. The insertion of the Cm-rpoN construction within the *ppcA *gene was verified by PCR and the resultant strain was named DLCN43.

In order to interrupt any of the two copies of the *rpoN *gene in DLCN43, a linear PCR fragment containing the *rpoN *gene disrupted by the kanamycin resistance cassette was constructed with cross-over PCR. The 5' region of *rpoN *was amplified with primer pair: RpoNKmII-1 and RpoNKmII-2. The 3' region of *rpoN *was amplified with RpoNKmII-5 and RpoNKmII-6. The Kan^R ^cassette was amplified with RpoNKmII-3 and RpoNKmII-4. The recombinant PCR was carried out as described in the previous section and the resultant recombinant PCR product was electroporated into the strain DLCN43. A total of 15 Kan^R ^transformants were isolated. However, all 15 transformants had the Kan^R ^insertion within the Cm-rpoN locus.

### Over-expression of *rpoN in trans *under the control of a *lac *or an IPTG-inducible *taclac *promoter

The complete *rpoN *coding sequence was amplified with primer sets RpoNfor-XbaI and RpoNrev-EcoRI for insertion to pJMG (*lac *promoter, gentamycin resistant) [66,67] or RpoNfor-EcoRI and RpoNrev-HindIII for insertion to pCD341 (*taclac *promoter, kanamycin resistant) [68] using Phusion High-Fidelity DNA polymerase (New England Biolabs) under the following conditions: 98°C, 30 s followed by 30 cycles of 98°C,20 s; 58°C, 20 s; 72°C, 60 s; and a final extension at 72°C for 10 min. The PCR product of the *rpoN *coding sequence was digested with restriction enzyme sets of *Xba *I and *Eco*R I or *Eco*R I and *Hind *III and inserted into the *Xba *I and *Eco*R I sites of the vector pJMG or the *Eco*R I and *Hind *III sites of the vector pCD341 via ligation; the resulting plasmids were designated pJMG rpoN or pCD rpoN, respectively. The *rpoN *gene in pJMGrpoN or pCDrpoN was then sequenced to screen for PCR artifacts.

Following electroporation of strain DL1 with pJMGrpoN or pCDrpoN, a gentamycin-resistant transformant or a kanamycin-resistant transformant, was isolated and designated DL1/pJMGrpoN or DL1/pCDrpoN (RpoN^+ ^for simplification), respectively. The presence of the plasmid in the DL1 strain was confirmed by plasmid purification and PCR.

The over-expression of *rpoN *for the strain containing pCDrpoN was achieved by adding 1 mM IPTG, a non-degradable analog of lactose to the medium. In the absence of lactose, transcription from the *taclac *promoter is inhibited by the *lacZ *repressor [69]. Upon addition of lactose or IPTG, the *lacZ *repressor is inactivated, therefore inducing transcription of the *rpoN *operon.

### Primer extension analyses

Total RNA was isolated from mid-exponential-phase cultures with RNeasy Midi kits (Qiagen) followed by treatment with RNase-free DNase (Ambion). Primer extension experiments were performed at 42°C using AMV reverse transcriptase (Roche) with primers GSU0364-06, GSU0420-04, GSU0777-04, GSU0938-06, GSU1836-04, GSU2005-02, GSU2302-04, GSU2490-02, GSU2751-02, GSU2806–08, GSU3046-02, and GSU3206-06, respectively for the corresponding promoter regions. The sequencing ladders presented in Figure 3 and Additional file 6 were also generated with these same primers using Thermo Sequenase Cycle sequencing kit (USB).

### DNA microarray hybridization and statistical analysis

DNA microarray hybridization was carried out as previously described [70]. Briefly, total RNA was extracted from three sets of identically treated batch cultures of the wild type harboring an empty vector (DL1/pCD341 or WT^V ^for simplification) and the RpoN overexpressing strains (RpoN^+^). Ten micrograms of RNA from the wild type and the RpoN^+ ^strain samples were chemically labeled with Cy3 or Cy5 fluorescent dyes respectively, using the MicroMax ASAP RNA Labeling Kit (Perkin Elmer), according to manufacturer's instructions. Labeled RNA was fragmented in a 20 μl volume at 70°C for 30 min using Ambion's Fragmentation Reagent and competitively hybridized to 12 K Arrays (Combimatrix) according to manufacturer's protocol. The arrays were scanned using a GenePix 4000B scanner (Molecular Devices), and analyzed using GenePix and Acuity 4.0 software. LIMMA mixed model analysis (R-package LIMMA [71]) was applied to the normalized Log_2 _expression ratios to identify differentially expressed genes. The P-value was then corrected for multiple comparisons according to Benjamini and Hochberg's procedure [72] to control the false discovery rate (FDR). Genes whose expression was significantly changed are listed in Additional files 1 &2 according to their fold changes (≤ -1.5 for down-regulation and ≥ +1.5 for up-regulation) and the P-values (≤ 0.0005). A gene was considered differentially expressed if at least half of its probes had a P ≤ 0.0005 and a fold change ≤ -1.5 or ≥ +1.5.

Gene expression microarray data (raw data and statistically processed data files) for the *G. sulfurreducens *over-expressing RpoN strain are available from the NCBI GEO (Gene Expression Omnibus) database http://www.ncbi.nlm.nih.gov/geo/, with accession GSE8022.

### Computational analysis of RpoN-regulated promoters and their target operons

RpoN-regulated promoters were predicted in the genome of *G. sulfurreducens *using the PromScan software [27]. This software assigned scores representing the Kullback-Leibler distance for predicted RpoN sites in the *G. sulfurreducens *genome, based on 186 known RpoN promoter sites from 47 bacterial species [2]. The predicted sequence elements were ranked according to their PromScan scores, and sequence elements with scores equal to or exceeding the default cutoff of 80 were selected for further consideration.

The operon organization of the *G. sulfurreducens *genome was predicted using a commercial version of the FGENESB software (V. Solovyev, A. Salamov, and P. Kosarev, unpublished; Softberry, Inc; 2003–2008). The reference June 1, 2004 version of operon annotation used in this study has been described previously [18]. For all RpoN-regulated promoter elements predicted by PromScan, we compared their genome location and strand orientation relative to operons and singleton ORFs. Those sequence elements that were located upstream of and in the same direction with protein-coding genes were considered to be possible RpoN-regulated promoter elements. Only those elements that did not overlap with coding genes (according to gene boundaries predicted by the FGENESB software) were selected for further consideration.

To compare the predicted locations of RpoN-regulated promoters with experimental evidence, we identified predicted RpoN promoters located upstream of and in the same orientation with genes with significantly altered expression in the RpoN^+ ^strain. This was achieved by comparing the list of suggested target genes located downstream of RpoN promoters (see Additional file 4) to the list of genes with significantly altered expression in the RpoN^+ ^strain and identifying the genes present in both lists.

Consensus sequences of predicted RpoN promoters was computed using our software, CONSENS by J. Krushkal [73]. Each nucleotide reported in the output consensus sequence represents the most frequent nucleotide. For ambiguous nucleotides co-occurring with equal highest frequencies, degenerate symbols were used according to the IUPAC-IUB ambiguity codes. Sequence logos of the predicted promoter sites were drawn using the WebLogo package v. 3 beta at http://weblogo.berkeley.edu/[74].

### *In silico *analysis of *G. sulfurreducens *growth

*In silico *modeling was utilized to analyze the possible phenotypes of *G. sulfurreducens *mutants in which genes encoding enzymes for ammonia assimilation pathway were deleted. The constraint-based genome-scale metabolic model of *G. sulfurreducens *[16] was applied in simulating cell growth using flux balance analysis and linear optimization [75] in SimPheny (Genomatica, Inc., CA). Biomass synthesis was selected as the objective function to be maximized in growth simulations. The following external metabolites were allowed to freely enter and leave the network for simulations of anaerobic growth on minimal media: Ca^2+^, CO_2_, Fe^2+^, H^+^, H_2_O, K^+^, Mg^2+^, Na^+^, NH^4+^, PO_4_^3-^, and SO_4_^2-^. Acetate was supplied to the metabolic model as electron donor and Fe(III) or fumarate was supplied as electron acceptor for the simulations. All other external metabolites were only allowed to leave the system.

### Preparation of antisera against RpoN

The *rpoN *coding region was amplified with primers pGEXrpoNEcoRIfor and pGEXrpoNXhoIrev, digested with *Eco*R I and *Xho *I, and inserted into the *Eco*R I-*Xho *I sites of pGEX-4T-1 (GE). Competent *E. coli *strain JM109 was transformed with the resulting plasmid, pGEXrpoN. The *E. coli *cell lysates containing the over-expressed GST-tagged RpoN was size-fractioned by sodium dodecyl sulfate-polyacrylamide gel electrophoresis. The band corresponding to RpoN protein was cut, crushed, and used to immunize New Zealand rabbits for antibody production against RpoN as described by Harlow and Lane [76].

### Analytical techniques

Protein concentration was determined using the bicinchoninic acid method with bovine serum albumin as a standard [77]. Western blot analyses were carried out by using antiserum against RpoN according to the protocol described by Ausubel *et al *[78]. Immunoreactive bands were visualized using an alkaline phosphatase-conjugated goat anti-rabbit secondary antibody (Pierce) and 1-step NBT/BCIP plus suppressor (Pierce) according to the manufacturer's instructions. Growth of fumarate cultures was monitored by measuring turbidity at 600 nm in a Genesys 2 spectrophotometer (Spectronic Instruments). Cell density of Fe(III)-grown cultures were determined using epifluorescence microscopy with acridine orange staining [79]. Fe(II) concentrations were determined with the ferrozine assay as previously described [80]. Agglutination assays were preformed as described by Reguera *et al *[31].

## Authors' contributions

CL designed this study, carried out experimental work, drafted the manuscript and interpreted microarray analysis and PromScan data. JK oversaw the computational prediction of RpoN-regulated promoters, interpreted results and drafted the manuscript. TU drafted the manuscript and carried out part of the primer extension assays. MP predicted RpoN-regulated promoter elements and localized them relative to gene expression changes and operon locations. JS performed *in silico *modeling analysis to predict the effects of GS, GOGAT and/or GDH deletion mutations on cell growth under different conditions. KJ immunized rabbits for antiserum production against RpoN. CN participated in RpoN mutagenesis. GR participated in agglutination analysis. RD and BP carried out microarray hybridization. RMA assisted with interpretation of RpoN promoter analysis and bioinformatics data integration. DRL designed the study and participated in interpretation of the results and drafted the manuscript. All authors participated in editing this manuscript and approved of its final form.

## Supplementary Material

Additional file 1 List of up-regulated genes in the RpoN over-expressing strain as compared to the wild type strain, based on fold change cutoff 1.5. Click here for file

Additional file 2 List of down-regulated genes in the RpoN over-expressing strain as compared to the wild type strain, based on fold change cutoff 1.5. Click here for file

Additional file 3 List of 110 predicted RpoN-regulated promoters located in the noncoding regions, upstream of and in the same orientation with protein-coding genes. Click here for file

Additional file 4 List of 110 predicted RpoN-regulated promoters and their target downstream genes. Click here for file

Additional file 5 List of operons/genes containing RpoN-dependent promoters identified by the PromScan analysis and also identified using transcriptome analysis. Click here for file

Additional file 6 **RpoN-dependent gene expression**. Representative genes, (a) GSU0364, GSU2005, GSU2302, GSU2490 and GSU3206 (up-regulated in the RpoN^+ ^strain), and (b) GSU0938 (down-regulated in the RpoN^+ ^strain) identified by the microarray analysis were analyzed by primer extension assays. The results of the primer extension assays and their promoter regions are shown. The 5' ends of mRNA are indicated by asterisks. RBS sites are underlined. Translation start codons are in bold and are indicated by Met. Click here for file

Additional file 7 Primers used in this work. Click here for file
