# Supplementary material for: Genome-wide analysis of the RpoN regulon in Geobacter sulfurreducens
Source: BMC Genomics. 2009 Jul 22;10:331. doi: 10.1186/1471-2164-10-331 (PMC2725144; doi:10.1186/1471-2164-10-331)
Supplement: Additional file 1 — List of up-regulated genes in the RpoN over-expressing strain as compared to the wild type strain, based on fold change cutoff 1.5. [file 1471-2164-10-331-S1.pdf]

Additional file 1. List of up-regulated genes in the RpoN over-expressing strain as compared to the wild type strain, based on fold change cutoff 1.5

Up-Regulated gene list

Name	Product	Main Role	Fold changes
GSU1279	cobalamin biosynthesis protein CbiM, putative	Biosynthesis of cofactors, prosthetic groups, and carriers	1.81
GSU1917	undecaprenyl diphosphate synthase	Biosynthesis of cofactors, prosthetic groups, and carriers	1.52
GSU0135	delta-aminolevulinic acid dehydratase	Biosynthesis of cofactors, prosthetic groups, and carriers	1.5
GSU0727	lipoprotein, putative	Cell envelope	2.27
GSU2089	rod shape-determining protein MreB	Cell envelope	1.87
GSU0667	membrane protein, putative	Cell envelope	1.79
GSU2302	trehalose-phosphatase, putative	Cellular processes	7.53
GSU1880	S-adenosylmethionine synthetase	Central intermediary metabolism	1.56
GSU0364	cytochrome c3 PpcB	Energy metabolism	2.78
GSU2504	cytochrome c family protein OmcS	Energy metabolism	2.6
GSU0466	cytochrome c551 peroxidase MacA	Energy metabolism	2
GSU1996	cytochrome c family protein	Energy metabolism	1.96
GSU1024	cytochrome c3 PpcD	Energy metabolism	1.82
GSU3274	cytochrome c family protein, putative	Energy metabolism	1.77
GSU0994	fumarate hydratase, class I	Energy metabolism	1.71
GSU1284	cytochrome c, putative	Energy metabolism	1.7
GSU0109	ATP synthase F0, B subunit	Energy metabolism	1.61
GSU2732	cytochrome c family protein	Energy metabolism	1.61
GSU0108	ATP synthase F0, B' subunit, putative	Energy metabolism	1.59
GSU3188	rubredoxin	Energy metabolism	1.59
GSU0112	ATP synthase F1, gamma subunit	Energy metabolism	1.59
GSU2503	cytochrome c family protein OmcT	Energy metabolism	1.57
GSU1305	Glu/Leu/Phe/Val dehydrogenase family protein	Energy metabolism	1.56
GSU0592	cytochrome c family protein	Energy metabolism	1.54
GSU2737	polyheme membrane-associated cytochrome c OmcB	Energy metabolism	1.53
GSU2513	cytochrome c family protein	Energy metabolism	1.51
GSU0111	ATP synthase F1, alpha subunit	Energy metabolism	1.5
GSU1402	acetyl-CoA carboxylase, carboxyl transferase, alpha subunit	Fatty acid and phospholipid metabolism	1.77
GSU2370	acetyl-CoA carboxylase, carboxyl transferase, beta subunit	Fatty acid and phospholipid metabolism	1.57
GSU1601	3-oxoacyl-(acyl-carrier-protein) synthase III	Fatty acid and phospholipid metabolism	1.55
GSU0723	hypothetical protein	Hypothetical proteins	2.86
GSU3467	conserved hypothetical protein TIGR00278	Hypothetical proteins	2.12
GSU3105	hypothetical protein	Hypothetical proteins	1.94
GSU0384	hypothetical protein	Hypothetical proteins	1.81
GSU3467.1	conserved hypothetical protein TIGR00278	Hypothetical proteins	1.76
GSU2518	hypothetical protein	Hypothetical proteins	1.71
GSU3106	hypothetical protein	Hypothetical proteins	1.7

GSU3289	hypothetical protein	Hypothetical proteins	1.67
GSU1771	hypothetical protein	Hypothetical proteins	1.54
GSU1278	hypothetical protein	Hypothetical proteins	1.53
GSU0450	hypothetical protein	Hypothetical proteins	1.52
GSU3410	hypothetical protein	No Data	3.31
GSU0468	hypothetical protein	No Data	3.2
GSU0722	hypothetical protein	No Data	2.86
GSU0595b	No Data	No Data	2.56
GSU2407	hypothetical protein	No Data	2.48
GSU1876	hypothetical protein	No Data	2.28
GSU1994	hypothetical protein	No Data	1.9
GSU2496	hypothetical protein	No Data	1.74
GSU2733	hypothetical protein	No Data	1.71
GSU0336	hypothetical protein	No Data	1.67
GSU2739	hypothetical protein	No Data	1.67
GSU2077	hypothetical protein	No Data	1.65
GSU1949	hypothetical protein	No Data	1.56
GSU1366	hypothetical protein	No Data	1.52
GSU0335	hypothetical protein	No Data	1.51
GSU1412	hypothetical protein	No Data	1.5
GSU2390	heat shock protein HtpG	Protein fate	1.98
GSU1793	trigger factor	Protein fate	1.98
GSU0033	chaperone protein dnaK	Protein fate	1.94
GSU2408	heat shock protein, Hsp20 family	Protein fate	1.75
GSU1792	ATP-dependent Clp protease, proteolytic subunit ClpP	Protein fate	1.68
GSU3340	60 kDa chaperonin	Protein fate	1.67
GSU0034	chaperone protein dnaJ	Protein fate	1.66
GSU1627	preprotein translocase, SecG subunit	Protein fate	1.65
GSU3339	chaperonin, 10 kDa	Protein fate	1.63
GSU1610	efflux transporter, RND family, MFP subunit	Protein fate	1.57
GSU2869	preprotein translocase, SecE subunit, putative	Protein fate	1.54
GSU0658	ClpB protein	Protein fate	1.51
GSU1791	ATP-dependent Clp protease, ATP-binding subunit ClpX	Protein fate	1.51
GSU2876	ribosomal protein L13	Protein synthesis	2.21
GSU2875	ribosomal protein S9	Protein synthesis	2.09
GSU2861.1	ribosomal protein S12	Protein synthesis	1.89
GSU2206	ribosomal protein S20	Protein synthesis	1.85
GSU2853	ribosomal protein S19	Protein synthesis	1.82
GSU2234	ribosomal protein L28	Protein synthesis	1.79
GSU0665	ribosomal protein S6	Protein synthesis	1.78
GSU0662	ribosoma protein L25	Protein synthesis	1.76
GSU0668	ribosomal protein L9	Protein synthesis	1.76

GSU2861	ribosomal protein S7	Protein synthesis	1.68
GSU2854	ribosomal protein L2	Protein synthesis	1.68
GSU2855	ribosomal protein L23	Protein synthesis	1.67
GSU2857	ribosomal protein L3	Protein synthesis	1.66
GSU2858	ribosomal protein S10	Protein synthesis	1.65
GSU3236	ribosomal protein L21	Protein synthesis	1.63
GSU2866	ribosomal protein L1	Protein synthesis	1.63
GSU1752	translation elongation factor P	Protein synthesis	1.61
GSU0666	ribosomal protein S18	Protein synthesis	1.61
GSU2867	ribosomal protein L11	Protein synthesis	1.61
GSU2852	ribosomal protein L22	Protein synthesis	1.58
GSU2870	ribosomal protein L33	Protein synthesis	1.58
GSU2850	ribosomal protein L16	Protein synthesis	1.56
GSU2845	ribosomal protein L5	Protein synthesis	1.56
GSU1599	ribosomal protein L32	Protein synthesis	1.56
GSU1921	ribosomal protein S2	Protein synthesis	1.55
GSU2856	ribosomal protein L4	Protein synthesis	1.54
GSU2849	ribosomal protein L29	Protein synthesis	1.53
GSU2844	ribosomal protein S14	Protein synthesis	1.53
GSU2848	ribosomal protein S17	Protein synthesis	1.51
GSU3104	peptide chain release factor 1	Protein synthesis	1.51
GSU1920	translation elongation factor Ts	Protein synthesis	1.51
GSU2841	ribosomal protein L18	Protein synthesis	1.51
GSU1110	nucleoside diphosphate kinase	Purines, pyrimidines, nucleosides, and nucleotides	2.06
GSU2698	transcriptional regulator, TetR family	Regulatory functions	2.26
GSU2666	transcriptional regulator, TetR family	Regulatory functions	1.98
GSU1836	nitrogen regulatory protein P-II	Regulatory functions	1.77
GSU1521	integration host factor, alpha subunit	Regulatory functions	1.75
GSU3292	transcriptional regulator, Fur family	Regulatory functions	1.64
GSU1522	transcriptional regulator, putative	Regulatory functions	1.64
GSU0514	transcriptional regulator, IclR family	Regulatory functions	1.58
GSU3206	dnaK suppressor protein, putative	Regulatory functions	1.56
GSU2969	sensory box/GGDEF family protein	Regulatory functions	1.53
GSU2219	response regulator	Signal transduction	1.69
GSU1887	RNA polymerase sigma-54 factor	Transcription	6.61
GSU3089	RNA polymerase sigma factor RpoD	Transcription	2.08
GSU0655	RNA polymerase sigma-32 factor	Transcription	1.97
GSU0491	ATP-dependent RNA helicase RhlE	Transcription	1.83
GSU2862	DNA-directed RNA polymerase, beta prime subunit	Transcription	1.61
GSU2303	Na+/H+ antiporter family protein	Transport and binding proteins	3.42
GSU2697	multidrug resistance protein	Transport and binding proteins	2.45
GSU2696	AcrB/AcrD/AcrF family protein	Transport and binding proteins	2.23

GSU2665	efflux transporter, RND family, MFP subunit	Transport and binding proteins	2.16
GSU0496	efflux transporter, RND family, MFP subunit	Transport and binding proteins	1.95
GSU2751	C4-dicarboxylate transporter, anaerobic	Transport and binding proteins	1.63
GSU0534	Rrf2 family protein	Unknown function	1.91
GSU0174	acetyl-CoA hydrolase/transferase family protein	Unknown function	1.86
GSU0455	rare lipoprotein A domain protein	Unknown function	1.79
GSU1877	oxidoreductase, 2-nitropropane dioxygenase family	Unknown function	1.7
GSU2715	fibronectin type III domain protein	Unknown function	1.68
GSU1904	decarboxylase family protein	Unknown function	1.66
GSU0024	OmpA domain protein	Unknown function	1.66
GSU0023	TPR domain protein	Unknown function	1.61
GSU1233	phosphoesterase, putative	Unknown function	1.6
GSU1721	radical SAM domain protein	Unknown function	1.59
GSU0029	hydrolase, carbon-nitrogen family	Unknown function	1.58
GSU1925	transport-associated domain protein	Unknown function	1.56
GSU3091	CvpA family protein	Unknown function	1.52
GSU0490	acetyl-CoA hydrolase/transferase family protein	Unknown function	1.52
