# Supplementary material for: Genome-wide analysis of the RpoN regulon in Geobacter sulfurreducens
Source: BMC Genomics. 2009 Jul 22;10:331. doi: 10.1186/1471-2164-10-331 (PMC2725144; doi:10.1186/1471-2164-10-331)
Supplement: Additional file 2 — List of down-regulated genes in the RpoN over-expressing strain as compared to the wild type strain, based on fold change cutoff 1.5. [file 1471-2164-10-331-S2.pdf]

Additional file 2. List of down-regulated genes in the RpoN over-expressing strain as compared to the wild type strain, based on fold change cutoff 1.5

Down-regulated gene list

Name	Product	Main Role	Fold changes
GSU1561	penicillin-binding protein, 1A family	Cell envelope	1.93
GSU0428	lipoprotein, putative	Cell envelope	1.82
GSU0805	cytoplasmic membrane protein FsxA	Cell envelope	1.66
GSU2360	maltooligosyltrehalose synthase, putative	Cell envelope	1.65
GSU1853	membrane protein, putative	Cell envelope	1.63
GSU1549	lipoprotein, putative	Cell envelope	1.56
GSU0038	lipoprotein, putative	Cell envelope	1.55
GSU2326	outer membrane lipoprotein	Cell envelope	1.52
GSU2122	TraG family protein	Cellular processes	1.94
GSU0422	flagellar motor switch protein FliN	Cellular processes	1.93
GSU0425	flagellar biosynthesis protein FliR	Cellular processes	1.72
GSU2416	chemotaxis protein CheW	Cellular processes	1.71
GSU2555	melittin resistance protein, putative	Cellular processes	1.52
GSU3015	flagellin FlaG, putative	Cellular processes	1.51
GSU1300	methyl-accepting chemotaxis protein	Cellular processes	1.51
GSU1810	cell cycle protein MesJ, putative	Cellular processes	1.5
GSU1507	heptosyltransferase family protein, putative	Central intermediary metabolism	1.53
GSU1421	nuclease SbcCD, D subunit, putative	DNA metabolism	1.53
GSU3228	cytochrome c family protein	Energy metabolism	1.59
GSU2656	pyruvate dehydrogenase complex E2 component, dihydrolipoamide acetyltransferase	Energy metabolism	1.5
GSU0796	acyl-CoA thioester hydrolase, putative	Fatty acid and phospholipid metabolism	1.51
GSU3085	conserved hypothetical protein TIGR00486	Hypothetical proteins	1.57
GSU3251	hypothetical protein	Hypothetical proteins	1.56
GSU1513	hypothetical protein	Hypothetical proteins	1.51
GSU2471	group II intron, maturase	Mobile and extrachromosomal element functions	7.22
GSU2772	ISGsu3, transposase	Mobile and extrachromosomal element functions	1.53
GSU1447	hypothetical protein	No Data	2.43
GSU2142.1	No Data	No Data	2.2
GSU3227	hypothetical protein	No Data	1.82
GSU3034	hypothetical protein	No Data	1.81
GSU1226	hypothetical protein	No Data	1.7
GSU1330b	No Data	No Data	1.69
GSU2036	hypothetical protein	No Data	1.66
GSU0759	hypothetical protein	No Data	1.6
GSU2116	hypothetical protein	No Data	1.59
GSU0639	hypothetical protein	No Data	1.56
GSU0330	general secretion pathway protein C, putative	Protein fate	1.53
GSU2000	tRNA delta(2)-isopentenylpyrophosphate transferase	Protein synthesis	1.73
GSU2524	sigma-54 dependent DNA-binding response regulator	Signal transduction	2.61
GSU0255	sensory box histidine kinase	Signal transduction	1.9

GSU3263	response regulator	Signal transduction	1.68
GSU3252	sensor histidine kinase	Signal transduction	1.58
GSU0035	2',5' RNA ligase, putative	Transcription	1.93
GSU1723	mechanosensitive ion channel family protein	Transport and binding proteins	2.57
GSU1340	ABC transporter, permease protein	Transport and binding proteins	1.68
GSU1435	peptide ABC transporter, permease protein	Transport and binding proteins	1.68
GSU2339	monovalent cation/proton antiporter, MrpF/PhaF family	Transport and binding proteins	1.6
GSU1732	branched-chain amino acid ABC transporter, permease	Transport and binding proteins	1.53
GSU1349	sulfate ABC transporter, ATP-binding protein	Transport and binding proteins	1.53
GSU2769	metallo-beta-lactamase family protein	Unknown function	2.47
GSU1009	GTP-binding protein	Unknown function	2.15
GSU0314	general secretion protein E N-terminal domain protein	Unknown function	1.77
GSU1090	signal transduction protein-related protein	Unknown function	1.75
GSU0287	HD domain protein	Unknown function	1.66
GSU2543	polysaccharide deacetylase domain protein	Unknown function	1.62
GSU0754	fibronectin type III domain protein	Unknown function	1.56
GSU1354	plasmid stabilization system family protein	Unknown function	1.54
GSU2525	nitroreductase family protein	Unknown function	1.53
GSU1796	DHH family protein	Unknown function	1.51
