# Supplementary material for: Genome-wide analysis of the RpoN regulon in Geobacter sulfurreducens
Source: BMC Genomics. 2009 Jul 22;10:331. doi: 10.1186/1471-2164-10-331 (PMC2725144; doi:10.1186/1471-2164-10-331)
Supplement: Additional file 3 — List of 110 predicted RpoN-regulated promoters located in the noncoding regions, upstream of and in the same orientation with protein-coding genes. [file 1471-2164-10-331-S3.pdf]

Additional file 3. List of 110 predicted RpoN-regulated promoters located in the noncoding regions, upstream of and in the same orientation with protein-coding genes. Sequence elements are sorted according to their PromScan scores.

Predicted promoter	PromScan score	Start position	End position	Strand	Sequence
Promoter 1	93	3359799	3359815	-	CTGGCACAACGGTTGCA
Promoter 2	93	1006976	1006992	+	CTGGCACGGCTCGTGCT
Promoter 3	92	439442	439458	+	CTGGTACGGCTTTTGCT
Promoter 4	92	3532151	3532167	+	ATGGCACCGGCGTTGCT
Promoter 5	92	2198355	2198371	+	ATGGCACTGTAGTTGCT
Promoter 6	92	1149442	1149458	+	TTGGCATGGGCGTTGCT
Promoter 7	91	3213663	3213679	-	TTGGCACCATAATTGCT
Promoter 8	91	3018461	3018477	-	TTGGCCCGCCTTTTGCT
Promoter 9	91	2120774	2120790	+	CTGGCACCCTAGTTGCT
Promoter 10	91	1008384	1008400	+	GTGGCACGGCCTATGCT
Promoter 11	90	834869	834885	+	TTGGCACAATGCTTGCC
Promoter 12	90	3751277	3751293	+	GTGGCACGGTCACTGCT
Promoter 13	90	3237711	3237727	-	GTGGCACTTTTGTTGCA
Promoter 14	90	1444460	1444476	+	TTGGCCCGCATATTGCT
Promoter 15	89	650815	650831	+	TTGGCAATGCTCTTGCA
Promoter 16	89	532879	532895	-	ATGGCATATATGTTGCT
Promoter 17	88	897407	897423	-	CTGGCACAGGCCGTGCT
Promoter 18	88	2905847	2905863	-	ATGGCCCCGCTTTTGCT
Promoter 19	88	2417127	2417143	-	GTGGCAAGGTTATTGCG
Promoter 20	88	1342606	1342622	-	CAGGCACAGCCTTTGCT
Promoter 21	88	1325893	1325909	-	TTGGCAGAGAGGTTGCT
Promoter 22	87	437081	437097	+	ACGGCACGGCTTTTGCC
Promoter 23	87	2210873	2210889	-	TTGGCATATGAATTGCT
Promoter 24	87	1465280	1465296	+	CTGGCACGCCAATTGGA
Promoter 25	86	451031	451047	+	CTGGCATTTCGGTTGCA
Promoter 26	86	3555926	3555942	+	GTGGCATGCTCCATGCT
Promoter 27	86	3483840	3483856	+	ATGGCAAAGCAATTGCG
Promoter 28	86	3350126	3350142	-	TTGGCACATAACATGCT
Promoter 29	86	3311556	3311572	+	TCGGCACGTAGGTTGCA
Promoter 30	86	3105042	3105058	-	TTGGCATGGACGGTGCT
Promoter 31	86	3087161	3087177	-	TTGGCACGTGGGGTGCA
Promoter 32	86	2892594	2892610	+	TTGGCAAATGAATTGCT
Promoter 33	86	2845669	2845685	-	TTGGTATGCCCATTGCT
Promoter 34	86	2715212	2715228	+	ATGGCGCAATCTTTGCA
Promoter 35	86	113456	113472	+	TTGGCCCTGATGTTGCA
Promoter 36	85	985792	985808	-	ATGGTACGGCTACTGCA
Promoter 37	85	954365	954381	+	CTGGCACGAATATCGCC
Promoter 38	85	3533112	3533128	+	CCGGCACTACTGTTGCA
Promoter 39	85	3494323	3494339	+	ATGGCAAAGACGTTGCG
Promoter 40	85	3206537	3206553	-	TCGGCACCTCTTTTGCT
Promoter 41	85	2765711	2765727	-	CTGGCATGGATATTGTT
Promoter 42	85	2735628	2735644	+	ATGGTATGCAAGTTGCT
Promoter 43	85	265714	265730	+	TTGGCCCGGTTCGTGCT
Promoter 44	85	2234938	2234954	-	ATGGCGTGAAAATTGCT
Promoter 45	85	1642205	1642221	+	TTGGTTCGGCTTTTGCT
Promoter 46	85	1350361	1350377	+	ATGGCATGTTTTCTGCT
Promoter 47	84	654563	654579	+	TTGGCGTGCTGGTTGCA
Promoter 48	84	389663	389679	-	TTGGTACGCAACCTGCT
Promoter 49	84	3261819	3261835	-	CCGGCAGGGCGATTGCT
Promoter 50	84	3169000	3169016	-	TTGGCTTGTTTGTTGCA

Promoter 51	84	2620522	2620538	-	ATGGAACATTGTTTGCA
Promoter 52	84	2030482	2030498	-	CTGGCACCGCTGTTTCA
Promoter 53	84	1876861	1876877	-	CTGGCCCGTGTGTTGCC
Promoter 54	84	1582569	1582585	-	ATGGCACTGAAATGGCA
Promoter 55	84	1167017	1167033	+	CTGGCCTGCTAATTGCA
Promoter 56	84	1081498	1081514	+	ATGGTATGCGGTTTGCA
Promoter 57	84	1011401	1011417	+	CTGGCATGCCTCGTGCC
Promoter 58	83	999145	999161	+	TTGGTACCTTGTTTGCA
Promoter 59	83	619135	619151	-	TTGACACTATTTTTGCA
Promoter 60	83	3697242	3697258	+	CTGGCATGTATATGGCA
Promoter 61	83	3512615	3512631	+	CAGGCCCGCTTTTTGCT
Promoter 62	83	3194370	3194386	-	AAGGCTCGGTTTTTGCT
Promoter 63	83	3093435	3093451	+	AAGGCACGGAACATGCA
Promoter 64	83	3027520	3027536	-	TTGGTATCCCCCTTGCT
Promoter 65	83	2765786	2765802	+	ATGGCACTCAACTTGAA
Promoter 66	83	2129841	2129857	-	GTGGTACGTATTATGCT
Promoter 67	83	197880	197896	-	ACGGCACGACAACTGCT
Promoter 68	83	1397366	1397382	+	TTGGCATTGCAGCTGCT
Promoter 69	83	1039939	1039955	+	TTGGTATACAGGTTGCT
Promoter 70	82	860308	860324	-	CCGGCAAGATCATTGCA
Promoter 71	82	3072482	3072498	+	CTGGCAAGTGCCCTGCA
Promoter 72	82	3005233	3005249	+	AGGGCATCGATATTGCT
Promoter 73	82	2798300	2798316	-	TTCGCCCGACTATTGCT
Promoter 74	82	1651315	1651331	+	GTGGCGCTTGAGTTGCA
Promoter 75	82	1455058	1455074	+	ATGGCACGGCCTGTGTA
Promoter 76	81	773999	774015	+	TTGGCACGGGACATGAA
Promoter 77	81	744982	744998	+	TTGGTATTCAATTTGCT
Promoter 78	81	628383	628399	-	CTGGCATACGGGGTGCA
Promoter 79	81	555596	555612	-	TTGACACGCTTTGTGCT
Promoter 80	81	465979	465995	-	CGGGCACCGCGGTTGCC
Promoter 81	81	3546239	3546255	-	ACGGCAGGTAAATTGCT
Promoter 82	81	3503117	3503133	+	ACGGAACACTTCTTGCT
Promoter 83	81	3179817	3179833	-	CTGGTATATTCTTTGCT
Promoter 84	81	3105888	3105904	-	TTGGAACACTTTATGCT
Promoter 85	81	3079447	3079463	-	GTGGCACGCTTAGTGTT
Promoter 86	81	2842541	2842557	-	TTGGCATCCTGCCTGCT
Promoter 87	81	2784628	2784644	-	TTGGCATGTAAATTGTA
Promoter 88	81	2597007	2597023	-	CTGGCACATATCTTACG
Promoter 89	81	1651206	1651222	+	CTGGCATCGTCACTGCA
Promoter 90	81	1523169	1523185	-	GTGGCAAAGGCGCTGCA
Promoter 91	81	1353732	1353748	+	CTGGCATTGTTTTTGAT
Promoter 92	81	1329103	1329119	+	TTGGCTTCAGTATTGCA
Promoter 93	81	1121057	1121073	-	GCGGCACGAGATCTGCA
Promoter 94	80	87167	87183	-	ATGGCACGTTTTATGAA
Promoter 95	80	854282	854298	-	ATGCCTCGCCGCTTGCA
Promoter 96	80	849091	849107	-	CTGGCATTATTTTTGTT
Promoter 97	80	716820	716836	+	TTGGCACGAATTTTTTT
Promoter 98	80	3462928	3462944	+	ACGGCACCGGCATTGCC
Promoter 99	80	2699909	2699925	+	ACGGGACGGGGATTGCA
Promoter 100	80	2247542	2247558	-	AAGGCCTGAATCTTGCT
Promoter 101	80	2186329	2186345	+	TGGGCACAACAAATGCT
Promoter 102	80	2135268	2135284	-	ATGGCAAATATTTAGCT
Promoter 103	80	2002713	2002729	-	TAGGCAGACTATTTGCT
Promoter 104	80	191151	191167	+	GTGGCACCGGGTTGGCG

Promoter 105	80	1874885	1874901	+	AGGGCAAGCTACTTGCG
Promoter 106	80	1843205	1843221	+	AAGGCACTGTTCCTGCA
Promoter 107	80	181724	181740	+	TTGGAATTCGAGTTGCT
Promoter 108	80	1540183	1540199	+	GTCGCCCGCACGTTGCT
Promoter 109	80	1380900	1380916	-	TCGGCACGCATTTTGTA
Promoter 110	80	1293612	1293628	-	ATGGCATGCAAATGGCC
