# Supplementary material for: Genome-wide analysis of the RpoN regulon in Geobacter sulfurreducens
Source: BMC Genomics. 2009 Jul 22;10:331. doi: 10.1186/1471-2164-10-331 (PMC2725144; doi:10.1186/1471-2164-10-331)
Supplement: Additional file 4 — List of 110 predicted RpoN-regulated promoters and their target downstream genes. [file 1471-2164-10-331-S4.pdf]

Additional file 4. List of 110 predicted RpoN-regulated promoters and their target downstream genes.

Operon organization of downstream genes is listed according to the operon annotation described by Krushkal et al. (2007; Functional and Integrative Genomics 7, 229-255).
Predicted operon organization is presented as operon number in the genome-gene number in the operon. For example, **1346 Op 1** represents gene 1 in operon 1346; **1346 Op 2** represents gene 2 in Operon 1346, etc.
Tu denotes singleton open reading frames that were not predicted to be co-transcribed with other genes.
GSU, G. sulfurreducens gene identifier. **COG**, Clusters of Orthologous Group database identifier.

Predicted site	PromScan score	Start	End	Strand	Sequence
Promoter 1	93	3359799	3359815	-	CTGGCACAACGGTTGCA

Operon/gene	Strand	Start	End	GSU	Gene	Annotation	Functional category	Functional subcategory	COG
1346 Op 1	-	3352242	3352952	GSU3050		flagella basal body P-ring formation protein flgA, putative	Cellular processes	Chemotaxis and motility	## COG1261
1346 Op 2	-	3352996	3353784	GSU3051	flgG-1	flagellar basal-body rod protein FlgG	Cellular processes	Chemotaxis and motility	## COG1749
1346 Op 3	-	3353883	3354611	GSU3052	flgG-2	flagellar basal-body rod protein FlgG	Cellular processes	Chemotaxis and motility	## COG1749
1346 Op 4	-	3354638	3355390	GSU3053	fliA	RNA polymerase sigma factor for flagellar Operon/gene/gene	Transcription	Transcription factors	## COG1191
1346 Op 5	-	3355387	3356316	GSU3054		ParA family protein	Cellular processes	Cell division	## COG0455
1346 Op 6	-	3356313	3357668	GSU3055		flagellar biosynthetic protein FlhF, putative	Cellular processes	Chemotaxis and motility	## COG1419
1346 Op 7	-	3357658	3359748	GSU3056	flhA	flagellar biosynthetic protein FlhA	Cellular processes	Chemotaxis and motility	## COG1298

Predicted site	PromScan score	Start	End	Strand	Sequence
Promoter 2	93	1006976	1006992	+	CTGGCACGGCTCGTGCT

Operon/gene	Strand	Start	End	GSU	Gene	Annotation	Functional category	Functional subcategory	COG
431 Tu 1	+	1007092	1008243	GSU0937	nifV	homocitrate synthase	Amino acid biosynthesis	Pyruvate family	## COG0119

Predicted site	PromScan score	Start	End	Strand	Sequence
Promoter 3	92	439442	439458	+	CTGGTACGGCTTTTGCT

Operon/gene	Strand	Start	End	GSU	Gene	Annotation	Functional category	Functional subcategory	COG
186 Op 1	+	439493	439912	GSU0407	flgB	flagellar basal-body rod protein FlgB	Cellular processes	Chemotaxis and motility	## COG1815
186 Op 2	+	439915	440355	GSU0408	flgC	flagellar basal-body rod protein FlgC	Cellular processes	Chemotaxis and motility	## COG1558

Predicted site	PromScan score	Start	End	Strand	Sequence
Promoter 4	92	3532151	3532167	+	ATGGCACCGGCGTTGCT

Operon/gene	Strand	Start	End	GSU	Gene	Annotation	Functional category	Functional subcategory	COG
1409 Tu 1	+	3532315	3533100	GSU3221		cytochrome c family protein	Energy metabolism	Electron transport	

Predicted site	PromScan score	Start	End	Strand	Sequence
Promoter 5	92	2198355	2198371	+	ATGGCACTGTAGTTGCT

Operon/gene	Strand	Start	End	GSU	Gene	Annotation	Functional category	Functional subcategory	COG
913 Op 1	+	2198520	2199674	GSU2005		branched-chain amino acid ABC transporter, periplasmic amino acid-b	Transport and binding proteins	Amino acids, peptides and amines	## COG0683
913 Op 2	+	2199786	2200652	GSU2006		branched-chain amino acid ABC transporter, permease protein	Transport and binding proteins	Amino acids, peptides and amines	## COG0559
913 Op 3	+	2200668	2201741	GSU2007		branched-chain amino acid ABC transporter, permease protein	Transport and binding proteins	Amino acids, peptides and amines	## COG0559
913 Op 4	+	2201752	2202471	GSU2008		branched-chain amino acid ABC transporter, ATP-binding protein	Transport and binding proteins	Amino acids, peptides and amines	## COG0411
913 Op 5	+	2202499	2203203	GSU2009		branched-chain amino acid ABC transporter, ATP-binding protein	Transport and binding proteins	Amino acids, peptides and amines	## COG0410
913 Op 6	+	2203228	2203662	GSU2010		CBS domain protein	Unknown function	General	## COG0517

Predicted site	PromScan score	Start	End	Strand	Sequence
Promoter 6	92	1149442	1149458	+	TTGGCATGGGCGTTGCT

Operon/gene	Strand	Start	End	GSU	Gene	Annotation	Functional category	Functional subcategory	COG
495 Op 1	+	1148603	1149289	GSU1062		cytochrome c, putative	Energy metabolism	Electron transport	
495 Op 2	+	1149545	1149943	GSU1063		hypothetical protein	Not available	Not available	

Predicted site	PromScan score	Start	End	Strand	Sequence
Promoter 7	91	3213663	3213679	-	TTGGCACCATAATTGCT

Operon/gene	Strand	Start	End	GSU	Gene	Annotation	Functional category	Functional subcategory	COG
1305 Op 1	-	3206802	3209942	GSU2912		cytochrome c family protein	Energy metabolism	Electron transport	
1305 Op 2	-	3209979	3210818	GSU2913		cytochrome c family protein	Energy metabolism	Electron transport	
1305 Op 3	-	3210885	3213479	GSU2914		NHL repeat protein	Unknown function	General	

Predicted site	PromScan score	Start	End	Strand	Sequence
Promoter 8	91	3018461	3018477	-	TTGGCCCGCCTTTTGCT

Operon/gene	Strand	Start	End	GSU	Gene	Annotation	Functional category	Functional subcategory	COG
1226 Tu 1	-	3017725	3018372	GSU2741		transcriptional regulator, TetR family	Regulatory functions	DNA interactions	## COG1309

Predicted site	PromScan score	Start	End	Strand	Sequence
Promoter 9	91	2120774	2120790	+	CTGGCACCCTAGTTGCT

Operon/gene	Strand	Start	End	GSU	Gene	Annotation	Functional category	Functional subcategory	COG
891 Tu 1	+	2120881	2123244	GSU1939		GAF domain/His Kinase A domain/HD domain protein	Unknown function	General	## COG2206

Predicted site	PromScan score	Start	End	Strand	Sequence
Promoter 10	91	1008384	1008400	+	GTGGCACGGCCTATGCT

Operon/gene	Strand	Start	End	GSU	Gene	Annotation	Functional category	Functional subcategory	COG
432 Op 1	+	1008591	1009343	GSU0938		hypothetical protein	Not available	Not available	
432 Op 2	+	1009395	1009733	GSU0939		nitrogen regulatory protein P-II, putative	Regulatory functions	Protein interactions	## COG0347
432 Op 3	+	1009771	1011240	GSU0940		ammonium transporter	Transport and binding proteins	Cations and iron carrying compounds	## COG0004

Predicted site	PromScan score	Start	End	Strand	Sequence
Promoter 11	90	834869	834885	+	TTGGCACAATGCTTGCC

Operon/gene	Strand	Start	End	GSU	Gene	Annotation	Functional category	Functional subcategory	COG
351 Op 1	+	834947	835519	GSU0777	fdnG	formate dehydrogenase, major subunit, selenocysteine-containing	Energy metabolism	Anaerobic	## COG0243
351 Op 2	+	835568	837979	GSU0777	fdnG	formate dehydrogenase, major subunit, selenocysteine-containing	Energy metabolism	Anaerobic	## COG0243
351 Op 3	+	837976	838809	GSU0778	fdnH	formate dehydrogenase, iron-sulfur subunit	Energy metabolism	Anaerobic	## COG0437
351 Op 4	+	838806	840011	GSU0779		formate dehydrogenase, b-type cytochrome subunit, putative	Energy metabolism	Anaerobic	
351 Op 5	+	840072	841508	GSU0780		formate dehydrogenase accessory protein FdhD	Energy metabolism	Anaerobic	## COG1526
351 Op 6	+	841535	841714	GSU0781		twin-arginine translocation protein, TatA/E family	Protein fate	Protein and peptide secretion and trafficking	

Predicted site	PromScan score	Start	End	Strand	Sequence
Promoter 12	90	3751277	3751293	+	GTGGCACGGTCACTGCT

Operon/gene	Strand	Start	End	GSU	Gene	Annotation	Functional category	Functional subcategory	COG
1499 Op 1	+	3751347	3751571	GSU3409		hypothetical protein	Not available	Not available	
1499 Op 2	+	3751637	3751867	GSU3410		hypothetical protein	Not available	Not available	

Predicted site	PromScan score	Start	End	Strand	Sequence
Promoter 13	90	3237711	3237727	-	GTGGCACTTTTGTTGCA

Operon/gene	Strand	Start	End	GSU	Gene	Annotation	Functional category	Functional subcategory	COG
1313 Op 1	-	3234566	3234880	GSU2938		hypothetical protein	Not available	Not available	
1313 Op 2	-	3234920	3236137	GSU2939		outer membrane porin FmdC, putative	Transport and binding proteins	Porins	
1313 Op 3	-	3236208	3237614	GSU2940		lipoprotein, putative	Cell envelope	Other	## COG2897

Predicted site	PromScan score	Start	End	Strand	Sequence
Promoter 14	90	1444460	1444476	+	TTGGCCCGCATATTGCT

Operon/gene	Strand	Start	End	GSU	Gene	Annotation	Functional category	Functional subcategory	COG
610 Tu 1	+	1444534	1444794	GSU1318		hypothetical protein	Not available	Not available	

Predicted site	PromScan score	Start	End	Strand	Sequence
Promoter 15	89	650815	650831	+	TTGGCAATGCTCTTGCA

Operon/gene	Strand	Start	End	GSU	Gene	Annotation	Functional category	Functional subcategory	COG
273 Op 1	+	650920	651933	GSU0615		cytochrome c family protein	Energy metabolism	Electron transport	
273 Op 2	+	651948	652940	GSU0616		cytochrome c family protein	Energy metabolism	Electron transport	
273 Op 3	+	652996	654081	GSU0617		NHL repeat domain protein	Unknown function	General	

Predicted site	PromScan score	Start	End	Strand	Sequence
Promoter 16	89	532879	532895	-	ATGGCATATATGTTGCT

Operon/gene	Strand	Start	End	GSU	Gene	Annotation	Functional category	Functional subcategory	COG
218 Op 1	-	532095	532700	GSU0499		M23/M37 peptidase domain protein	Unknown function	General	## COG0739
218 Op 2	-	532709	532840						

Predicted site	PromScan score	Start	End	Strand	Sequence
Promoter 17	88	897407	897423	-	CTGGCACAGGCCGTGCT

Operon/gene	Strand	Start	End	GSU	Gene	Annotation	Functional category	Functional subcategory	COG
377 Tu 1	-	896965	897363	GSU0840		hypothetical protein	Not available	Not available	

Predicted site	PromScan score	Start	End	Strand	Sequence
Promoter 18	88	2905847	2905863	-	ATGGCCCCGCTTTTGCT

Operon/gene	Strand	Start	End	GSU	Gene	Annotation	Functional category	Functional subcategory	COG
1178 Op 1	-	2905012	2905347	GSU2634		hypothetical protein	Not available	Not available	
1178 Op 2	-	2905385	2905804	GSU2635		hypothetical protein	Hypothetical proteins	Conserved	## COG2703
1178 Op 3	-	2905877	2907730	GSU2636		alpha-amylase family protein	Energy metabolism	Biosynthesis and degradation of polysaccharides	## COG0366
1178 Op 4	-	2907815	2908801	GSU2637		alcohol dehydrogenase, zinc-containing	Energy metabolism	Fermentation	## COG0604

Predicted site	PromScan score	Start	End	Strand	Sequence
Promoter 19	88	2417127	2417143	-	GTGGCAAGGTTATTGCG

Operon/gene	Strand	Start	End	GSU	Gene	Annotation	Functional category	Functional subcategory	COG
1001 Tu 1	-	2416007	2416930	GSU2202		transcriptional regulator, LysR family	Regulatory functions	DNA interactions	## COG0583

Predicted site	PromScan score	Start	End	Strand	Sequence
Promoter 20	88	1342606	1342622	-	CAGGCACAGCCTTTGCT

Operon/gene	Strand	Start	End	GSU	Gene	Annotation	Functional category	Functional subcategory	COG
576 Op 1	-	1337263	1338009	GSU1235		hypothetical protein	Hypothetical proteins	Conserved	## COG0070
576 Op 2	-	1338075	1339199	GSU1236		hypothetical protein	Hypothetical proteins	Conserved	## COG0067
576 Op 3	-	1339247	1340557	GSU1237		pyridine nucleotide-disulphide oxidoreductase family protein	Unknown function	Enzymes of unknown specificity	## COG0446
576 Op 4	-	1340554	1341003	GSU1238		iron-sulfur cluster-binding protein	Energy metabolism	Electron transport	## COG1142
576 Op 5	-	1341008	1342537	GSU1239		glutamate synthase-related protein	Unknown function	General	## COG0069

Predicted site	PromScan score	Start	End	Strand	Sequence
Promoter 21	88	1325893	1325909	-	TTGGCAGAGAGGTTGCT

Operon/gene	Strand	Start	End	GSU	Gene	Annotation	Functional category	Functional subcategory	COG
570 Tu 1	-	1324618	1325838	GSU1221		ammonium transporter, putative	Transport and binding proteins	Cations and iron carrying compounds	## COG0004

Predicted site	PromScan score	Start	End	Strand	Sequence
Promoter 22	87	437081	437097	+	ACGGCACGGCTTTTGCC

Operon/gene	Strand	Start	End	GSU	Gene	Annotation	Functional category	Functional subcategory	COG
185 Tu 1	+	437169	439259	GSU0406		TPR domain protein	Unknown function	General	

Predicted site	PromScan score	Start	End	Strand	Sequence
Promoter 23	87	2210873	2210889	-	TTGGCATATGAATTGCT

Operon/gene	Strand	Start	End	GSU	Gene	Annotation	Functional category	Functional subcategory	COG
917 Op 1	-	2208461	2210860	GSU2016		sensory box/GGDEF family protein	Regulatory functions	Small molecule interactions	## COG2200
917 Op 2	-	2210896	2211717	GSU2017		hypothetical protein	Hypothetical proteins	Conserved	## COG1427

Predicted site	PromScan score	Start	End	Strand	Sequence
Promoter 24	87	1465280	1465296	+	CTGGCACGCCAATTGGA

Operon/gene	Strand	Start	End	GSU	Gene	Annotation	Functional category	Functional subcategory	COG
617 Tu 1	+	1465350	1465754	GSU1338	Not avai	Not available	Not available	Not available	

Predicted site	PromScan score	Start	End	Strand	Sequence
Promoter 25	86	451031	451047	+	CTGGCATTTCGGTTGCA

Operon/gene	Strand	Start	End	GSU	Gene	Annotation	Functional category	Functional subcategory	COG
189 Op 1	+	451106	451627	GSU0420		flagellar protein FliL	Cellular processes	Chemotaxis and motility	## COG1580
189 Op 2	+	451662	452648	GSU0421	fliM	flagellar motor switch protein FliM	Cellular processes	Chemotaxis and motility	## COG1868
189 Op 3	+	452645	452950	GSU0422	fliN	flagellar motor switch protein FliN	Cellular processes	Chemotaxis and motility	## COG1886
189 Op 4	+	452947	453444	GSU0422.1	fliN	flagellar motor switch protein FliN	Cellular processes	Chemotaxis and motility	
189 Op 5	+	453450	454217	GSU0423	fliP	flagellar biosynthetic protein FliP	Cellular processes	Chemotaxis and motility	## COG1338
189 Op 6	+	454230	454499	GSU0424	fliQ	flagellar biosynthetic protein FliQ	Cellular processes	Chemotaxis and motility	## COG1987
189 Op 7	+	454550	455344	GSU0425	fliR	flagellar biosynthesis protein FliR	Cellular processes	Chemotaxis and motility	## COG1684
189 Op 8	+	455349	456407	GSU0426	flhB	flagellar biosynthetic protein FlhB	Cellular processes	Chemotaxis and motility	## COG1377

Predicted site	PromScan score	Start	End	Strand	Sequence
Promoter 26	86	3555926	3555942	+	GTGGCATGCTCCATGCT

Operon/gene	Strand	Start	End	GSU	Gene	Annotation	Functional category	Functional subcategory	COG
1416 Tu 1	+	3556109	3556216	GSU3242		hypothetical protein	Not available	Not available	

Predicted site	PromScan score	Start	End	Strand	Sequence
Promoter 27	86	3483840	3483856	+	ATGGCAAAGCAATTGCG

Operon/gene	Strand	Start	End	GSU	Gene	Annotation	Functional category	Functional subcategory	COG
1391 Op 1	+	3483866	3484321	GSU3175		hypothetical protein	Not available	Not available	
1391 Op 2	+	3484361	3485293	GSU3176		lysM domain protein	Unknown function	General	
1391 Op 3	+	3485350	3487443	GSU3177		Rhs family protein	Unknown function	General	## COG3501
1391 Op 4	+	3487475	3487750	GSU3178		hypothetical protein	Not available	Not available	
1391 Op 5	+	3487734	3487979	GSU3179		hypothetical protein	Hypothetical proteins	Domain	

Predicted site	PromScan score	Start	End	Strand	Sequence
Promoter 28	86	3350126	3350142	-	TTGGCACATAACATGCT

Operon/gene	Strand	Start	End	GSU	Gene	Annotation	Functional category	Functional subcategory	COG
1343 Op 1	-	3345798	3346277	GSU3040		hypothetical protein	Hypothetical proteins	Conserved	## COG1699
1343 Op 2	-	3346282	3346500	GSU3041	csrA	carbon storage regulator	Regulatory functions	RNA interactions	## COG1551
1343 Op 3	-	3346545	3347441	GSU3042	flgL	flagellar hook-associated protein FlgL	Cellular processes	Chemotaxis and motility	## COG1344
1343 Op 4	-	3347451	3348884	GSU3043	flgK	flagellar hook-associated protein FlgK	Cellular processes	Chemotaxis and motility	## COG1256
1343 Op 5	-	3348886	3349377	GSU3044		hypothetical protein	Not available	Not available	
1343 Op 6	-	3349396	3349683	GSU3045	flgM	negative regulator of flagellin synthesis FlgM	Regulatory functions	DNA interactions	
1343 Op 7	-	3349686	3350066	GSU3046		flagellar protein FlgJ-like protein	Cellular processes	Chemotaxis and motility	

Predicted site	PromScan score	Start	End	Strand	Sequence
Promoter 29	86	3311556	3311572	+	TCGGCACGTAGGTTGCA

Operon/gene	Strand	Start	End	GSU	Gene	Annotation	Functional category	Functional subcategory	COG
1338 Op 1	+	3311632	3311826	GSU3017		hypothetical protein	Hypothetical proteins	Domain	## COG0673
1338 Op 2	+	3311879	3312637	GSU3018		hypothetical protein	Hypothetical proteins	Conserved	## COG0500
1338 Op 3	+	3312678	3314636	GSU3019		dehydrogenase, E1 component, alpha and beta subunits	Unknown function	Enzymes of unknown specificity	## COG1071
1338 Op 4	+	3314645	3315748	GSU3020		hexapeptide transferase family protein	Unknown function	Enzymes of unknown specificity	## COG0110
1338 Op 5	+	3315764	3316954	GSU3021		DegT/DnrJ/EryC1/StrS family protein	Unknown function	General	## COG0399
1338 Op 6	+	3316979	3320353	GSU3022		hypothetical protein	Not available	Not available	
1338 Op 7	+	3320365	3327555	GSU3023		glycosyl transferase, group 38354 family protein	Cell envelope	Biosynthesis and degradation of surface polysacchar	## COG0438
1338 Op 8	+	3327569	3329374	GSU3024		hypothetical protein	Not available	Not available	
1338 Op 9	+	3329371	3330924	GSU3025		heptosyltransferase family protein	Cell envelope	Biosynthesis and degradation of surface polysacchar	## COG0859
1338 Op 10	+	3330990	3331268	GSU3026		flagellar protein FlbD, putative	Cellular processes	Chemotaxis and motility	## COG1582
1338 Op 11	+	3331289	3332053	GSU3027		chemotaxis MotA protein	Cellular processes	Chemotaxis and motility	## COG1291
1338 Op 12	+	3332063	3332863	GSU3028	motB	chemotaxis MotB protein	Cellular processes	Chemotaxis and motility	## COG1360

Predicted site	PromScan score	Start	End	Strand	Sequence
Promoter 30	86	3105042	3105058	-	TTGGCATGGACGGTGCT

Operon/gene	Strand	Start	End	GSU	Gene	Annotation	Functional category	Functional subcategory	COG
1274 Tu 1	-	3104018	3104887	GSU2821	nifH	nitrogenase iron protein	Central intermediary metabolism	Nitrogen fixation	## COG1348

Predicted site	PromScan score	Start	End	Strand	Sequence
Promoter 31	86	3087161	3087177	-	TTGGCACGTGGGGTGCA

Operon/gene	Strand	Start	End	GSU	Gene	Annotation	Functional category	Functional subcategory	COG
1263 Op 1	-	3082277	3083074	GSU2802	dRAT	NAD(+)--dinitrogen-reductase ADP-D-ribosyltransferase	Central intermediary metabolism	Nitrogen fixation	
1263 Op 2	-	3083133	3083462	GSU2803		dinitrogenase iron-molybdenum cofactor family protein	Central intermediary metabolism	Nitrogen fixation	## COG0535
1263 Op 3	-	3083491	3083754	GSU2804		ferredoxin family protein	Energy metabolism	Electron transport	## COG1145
1263 Op 4	-	3083822	3084211	GSU2805	nifX	nitrogenase molybdenum-iron cofactor biosynthesis protein NifX	Central intermediary metabolism	Nitrogen fixation	
1263 Op 5	-	3084293	3087052	GSU2806	nifEN	nitrogenase molybdenum-iron cofactor biosynthesis protein NifEN	Central intermediary metabolism	Nitrogen fixation	## COG2710

Predicted site	PromScan score	Start	End	Strand	Sequence
Promoter 32	86	2892594	2892610	+	TTGGCAAATGAATTGCT

Operon/gene	Strand	Start	End	GSU	Gene	Annotation	Functional category	Functional subcategory	COG
1171 Tu 1	+	2892918	2895026	GSU2622		HAMP domain/GAF domain/HD domain protein	Unknown function	General	## COG2206

Predicted site	PromScan score	Start	End	Strand	Sequence
Promoter 33	86	2845669	2845685	-	TTGGTATGCCCATTGCT

Operon/gene	Strand	Start	End	GSU	Gene	Annotation	Functional category	Functional subcategory	COG
1151 Tu 1	-	2844288	2845451	GSU2582.1		hypothetical protein	Not available	Not available	

Predicted site	PromScan score	Start	End	Strand	Sequence
Promoter 34	86	2715212	2715228	+	ATGGCGCAATCTTTGCA

Operon/gene	Strand	Start	End	GSU	Gene	Annotation	Functional category	Functional subcategory	COG
1107 Op 1	+	2715279	2716979	GSU2476		TPR domain protein	Unknown function	General	## COG0457
1107 Op 2	+	2717042	2719636	GSU2477		TPR domain/radical SAM/B12 binding domain protein	Unknown function	General	## COG1032

Predicted site	PromScan score	Start	End	Strand	Sequence
Promoter 35	86	113456	113472	+	TTGGCCCTGATGTTGCA

Operon/gene	Strand	Start	End	GSU	Gene	Annotation	Functional category	Functional subcategory	COG
44 Op 1	+	113608	114099	GSU0098		MglB protein	Cellular processes	Chemotaxis and motility	
44 Op 2	+	114118	114705	GSU0099		MglA protein	Cellular processes	Chemotaxis and motility	## COG1100

Predicted site	PromScan score	Start	End	Strand	Sequence
Promoter 36	85	985792	985808	-	ATGGTACGGCTACTGCA

Operon/gene	Strand	Start	End	GSU	Gene	Annotation	Functional category	Functional subcategory	COG
421 Op 1	-	985247	985468	GSU0919		hypothetical protein	Not available	Not available	
421 Op 2	-	985503	985739	GSU0920.1		hypothetical protein	Not available	Not available	

Predicted site	PromScan score	Start	End	Strand	Sequence
Promoter 37	85	954365	954381	+	CTGGCACGAATATCGCC

Operon/gene	Strand	Start	End	GSU	Gene	Annotation	Functional category	Functional subcategory	COG
406 Tu 1	+	954472	956514	GSU0891.1		subtilase domain protein	Unknown function	General	## COG0210

Predicted site	PromScan score	Start	End	Strand	Sequence
Promoter 38	85	3533112	3533128	+	CCGGCACTACTGTTGCA

Operon/gene	Strand	Start	End	GSU	Gene	Annotation	Functional category	Functional subcategory	COG
1410 Op 1	+	3533249	3534487	GSU3222		NHL repeat domain protein	Unknown function	General	
1410 Op 2	+	3534527	3535096	GSU3223		cytochrome c family protein	Energy metabolism	Electron transport	
1410 Op 3	+	3535106	3537139	GSU3224		hypothetical protein	Not available	Not available	
1410 Op 4	+	3537164	3538261	GSU3225		NHL repeat domain protein	Unknown function	General	
1410 Op 5	+	3538273	3540003	GSU3226		cytochrome c family protein	Energy metabolism	Electron transport	

Predicted site	PromScan score	Start	End	Strand	Sequence
Promoter 39	85	3494323	3494339	+	ATGGCAAAGACGTTGCG

Operon/gene	Strand	Start	End	GSU	Gene	Annotation	Functional category	Functional subcategory	COG
1394 Tu 1	+	3494464	3495471	GSU3186		hypothetical protein	Hypothetical proteins	Conserved	

Predicted site	PromScan score	Start	End	Strand	Sequence
Promoter 40	85	3206537	3206553	-	TCGGCACCTCTTTTGCT

Operon/gene	Strand	Start	End	GSU	Gene	Annotation	Functional category	Functional subcategory	COG
1304 Op 1	-	3201779	3202648	GSU2908		hypothetical protein	Not available	Not available	
1304 Op 2	-	3202707	3203264	GSU2909		lipoprotein, putative	Cell envelope	Other	
1304 Op 3	-	3203337	3204026	GSU2910		hypothetical protein	Not available	Not available	
1304 Op 4	-	3204023	3206512	GSU2911		hypothetical protein	Not available	Not available	

Predicted site	PromScan score	Start	End	Strand	Sequence
Promoter 41	85	2765711	2765727	-	CTGGCATGGATATTGTT

Operon/gene	Strand	Start	End	GSU	Gene	Annotation	Functional category	Functional subcategory	COG
1121 Op 1	-	2762042	2763736	GSU2506		sensory box protein/sigma-54 dependent DNA-binding response regula	Signal transduction	Two-component systems	## COG3829
1121 Op 2	-	2763705	2765570	GSU2507		sensor histidine kinase	Signal transduction	Two-component systems	## COG0642

Predicted site	PromScan score	Start	End	Strand	Sequence
Promoter 42	85	2735628	2735644	+	ATGGTATGCAAGTTGCT

Operon/gene	Strand	Start	End	GSU	Gene	Annotation	Functional category	Functional subcategory	COG
1114 Tu 1	+	2735710	2737077	GSU2490		oxalate/formate antiporter, putative	Transport and binding proteins	Carbohydrates, organic alcohols, and acids	## COG0477

Predicted site	PromScan score	Start	End	Strand	Sequence
Promoter 43	85	265714	265730	+	TTGGCCCGGTTCGTGCT

Operon/gene	Strand	Start	End	GSU	Gene	Annotation	Functional category	Functional subcategory	COG
119 Tu 1	+	265796	266233	GSU0256		hypothetical protein	Hypothetical proteins	Conserved	## COG2703

Predicted site	PromScan score	Start	End	Strand	Sequence
Promoter 44	85	2234938	2234954	-	ATGGCGTGAAAATTGCT

Operon/gene	Strand	Start	End	GSU	Gene	Annotation	Functional category	Functional subcategory	COG
922 Op 1	-	2226192	2226845	GSU2034		hypothetical protein	Not available	Not available	
922 Op 2	-	2226863	2228044	GSU2035		hypothetical protein	Not available	Not available	
922 Op 3	-	2228053	2228442	GSU2036		hypothetical protein	Not available	Not available	
922 Op 4	-	2228420	2228905	GSU2037		hypothetical protein	Not available	Not available	
922 Op 5	-	2228907	2234492	GSU2038		hypothetical protein	Not available	Not available	## COG3419
922 Op 6	-	2234591	2234893	GSU2039		hypothetical protein	Not available	Not available	

Predicted site	PromScan score	Start	End	Strand	Sequence
Promoter 45	85	1642205	1642221	+	TTGGTTCGGCTTTTGCT

Operon/gene	Strand	Start	End	GSU	Gene	Annotation	Functional category	Functional subcategory	COG
688 Op 1	+	1642314	1642529	GSU1496		pilin domain protein	Unknown function	General	## COG2165
688 Op 2	+	1642557	1642931	GSU1497		hypothetical protein	Not available	Not available	

Predicted site	PromScan score	Start	End	Strand	Sequence
Promoter 46	85	1350361	1350377	+	ATGGCATGTTTTCTGCT

Operon/gene	Strand	Start	End	GSU	Gene	Annotation	Functional category	Functional subcategory	COG
582 Op 1	+	1350455	1352029	GSU1249		sensory box histidine kinase	Signal transduction	Two-component systems	## COG0642
582 Op 2	+	1352057	1353466	GSU1250		sigma-54 dependent DNA-binding response regulator	Signal transduction	Two-component systems	## COG2204

Predicted site	PromScan score	Start	End	Strand	Sequence
Promoter 47	84	654563	654579	+	TTGGCGTGCTGGTTGCA

Operon/gene	Strand	Start	End	GSU	Gene	Annotation	Functional category	Functional subcategory	COG
274 Tu 1	+	654657	655355	GSU0618		cytochrome c family protein	Energy metabolism	Electron transport	

Predicted site	PromScan score	Start	End	Strand	Sequence
Promoter 48	84	389663	389679	-	TTGGTACGCAACCTGCT

Operon/gene	Strand	Start	End	GSU	Gene	Annotation	Functional category	Functional subcategory	COG
160 Op 1	-	387351	388952	GSU0357		cytochrome c family protein	Energy metabolism	Electron transport	## COG3303
160 Op 2	-	388985	389488	GSU0358		iron-sulfur cluster-binding protein	Energy metabolism	Electron transport	## COG1145

Predicted site	PromScan score	Start	End	Strand	Sequence
Promoter 49	84	3261819	3261835	-	CCGGCAGGGCGATTGCT

Operon/gene	Strand	Start	End	GSU	Gene	Annotation	Functional category	Functional subcategory	COG
1321 Tu 1	-	3261189	3261737	GSU2965		hypothetical protein	Hypothetical proteins	Conserved	

Predicted site	PromScan score	Start	End	Strand	Sequence
Promoter 50	84	3169000	3169016	-	TTGGCTTGTTTGTTGCA

Operon/gene	Strand	Start	End	GSU	Gene	Annotation	Functional category	Functional subcategory	COG
1293 Op 1	-	3163008	3166124	GSU2884		cytochrome c family protein	Energy metabolism	Electron transport	
1293 Op 2	-	3166135	3168801	GSU2885		NHL repeat domain protein	Unknown function	General	

Predicted site	PromScan score	Start	End	Strand	Sequence
Promoter 51	84	2620522	2620538	-	ATGGAACATTGTTTGCA

Operon/gene	Strand	Start	End	GSU	Gene	Annotation	Functional category	Functional subcategory	COG
1058 Op 1	-	2606832	2607443	GSU2378	trpF	N-(5'phosphoribosyl)anthranilate isomerase	Amino acid biosynthesis	Aromatic amino acid family	## COG0135
1058 Op 2	-	2607448	2608803	GSU2379		pyridoxal-phosphate dependent enzyme	Amino acid biosynthesis	Aromatic amino acid family	## COG1350
1058 Op 3	-	2608851	2609651	GSU2380	trpC	Indole-3-glycerol phosphate synthase	Amino acid biosynthesis	Aromatic amino acid family	## COG0134
1058 Op 4	-	2609656	2610708	GSU2381	trpD	anthranilate phosphoribosyltransferase	Amino acid biosynthesis	Aromatic amino acid family	## COG0547
1058 Op 5	-	2610773	2611345	GSU2382	trpG	anthranilate synthase component II	Amino acid biosynthesis	Aromatic amino acid family	## COG0512
1058 Op 6	-	2611347	2612822	GSU2383	trpE	anthranilate synthase component I	Amino acid biosynthesis	Aromatic amino acid family	## COG0147
1058 Op 7	-	2612913	2614757	GSU2384		sensor histidine kinase	Signal transduction	Two-component systems	## COG0642
1058 Op 8	-	2614738	2615343	GSU2385		hypothetical protein	Not available	Not available	
1058 Op 9	-	2615333	2616358	GSU2386		methylcobamide:CoM methyltransferase-related protein	Unknown function	General	
1058 Op 10	-	2616355	2617035	GSU2387		B12-binding domain	Unknown function	General	## COG1410
1058 Op 11	-	2617037	2619238	GSU2388		sensory box histidine kinase	Signal transduction	Two-component systems	## COG0642
1058 Op 12	-	2619301	2620449	GSU2389		ABC transporter, periplasmic substrate-binding protein, putative	Transport and binding proteins	Unknown substrate	## COG0683

Predicted site	PromScan score	Start	End	Strand	Sequence
Promoter 52	84	2030482	2030498	-	CTGGCACCGCTGTTTCA

Operon/gene	Strand	Start	End	GSU	Gene	Annotation	Functional category	Functional subcategory	COG
858 Tu 1	-	2030029	2030142	GSU1855.2		capsule polysaccharide export protein, putative	Transport and binding proteins	Other	

Predicted site	PromScan score	Start	End	Strand	Sequence
Promoter 53	84	1876861	1876877	-	CTGGCCCGTGTGTTGCC

Operon/gene	Strand	Start	End	GSU	Gene	Annotation	Functional category	Functional subcategory	COG
793 Tu 1	-	1876494	1876634						

Predicted site	PromScan score	Start	End	Strand	Sequence
Promoter 54	84	1582569	1582585	-	ATGGCACTGAAATGGCA

Operon/gene	Strand	Start	End	GSU	Gene	Annotation	Functional category	Functional subcategory	COG
671 Op 1	-	1579416	1581380	GSU1443		sensor histidine kinase/response regulator	Signal transduction	Two-component systems	## COG0642
671 Op 2	-	1581389	1582282	GSU1444		hypothetical protein	Hypothetical proteins	Conserved	## COG2984
671 Op 3	-	1582289	1582393						

Predicted site	PromScan score	Start	End	Strand	Sequence
Promoter 55	84	1167017	1167033	+	CTGGCCTGCTAATTGCA

Operon/gene	Strand	Start	End	GSU	Gene	Annotation	Functional category	Functional subcategory	COG
504 Tu 1	+	1167082	1167798	GSU1079		hypothetical protein	Not available	Not available	

Predicted site	PromScan score	Start	End	Strand	Sequence
Promoter 56	84	1081498	1081514	+	ATGGTATGCGGTTTGCA

Operon/gene	Strand	Start	End	GSU	Gene	Annotation	Functional category	Functional subcategory	COG
462 Tu 1	+	1081683	1082066	GSU1001		hypothetical protein	Not available	Not available	

Predicted site	PromScan score	Start	End	Strand	Sequence
Promoter 57	84	1011401	1011417	+	CTGGCATGCCTCGTGCC

Operon/gene	Strand	Start	End	GSU	Gene	Annotation	Functional category	Functional subcategory	COG
433 Tu 1	+	1011471	1012217	GSU0941		sensor histidine kinase, putative	Signal transduction	Two-component systems	## COG0642

Predicted site	PromScan score	Start	End	Strand	Sequence
Promoter 58	83	999145	999161	+	TTGGTACCTTGTTTGCA

Operon/gene	Strand	Start	End	GSU	Gene	Annotation	Functional category	Functional subcategory	COG
425 Tu 1	+	999205	999729	GSU0930		sulfur transferase, putative, selenocysteine-containing	Unknown function	General	

Predicted site	PromScan score	Start	End	Strand	Sequence
Promoter 59	83	619135	619151	-	TTGACACTATTTTTGCA

Operon/gene	Strand	Start	End	GSU	Gene	Annotation	Functional category	Functional subcategory	COG
258 Tu 1	-	617517	618791	GSU0584		hypothetical protein	Not available	Not available	

Predicted site	PromScan score	Start	End	Strand	Sequence
Promoter 60	83	3697242	3697258	+	CTGGCATGTATATGGCA

Operon/gene	Strand	Start	End	GSU	Gene	Annotation	Functional category	Functional subcategory	COG
1478 Tu 1	+	3697317	3697505	GSU3364		hypothetical protein	Not available	Not available	

Predicted site	PromScan score	Start	End	Strand	Sequence
Promoter 61	83	3512615	3512631	+	CAGGCCCCGCTTTTTGCT

Operon/gene	Strand	Start	End	GSU	Gene	Annotation	Functional category	Functional subcategory	COG
1402 Tu 1	+	3512703	3513122	GSU3206		dnaK suppressor protein, putative	Regulatory functions	DNA interactions	## COG1734

Predicted site	PromScan score	Start	End	Strand	Sequence
Promoter 62	83	3194370	3194386	-	AAGGCTCGGTTTTTGCT

Operon/gene	Strand	Start	End	GSU	Gene	Annotation	Functional category	Functional subcategory	COG
1302 Op 1	-	3182745	3191156	GSU2898		high-molecular-weight cytochrome c	Energy metabolism	Electron transport	
1302 Op 2	-	3191201	3194140	GSU2899		high-molecular-weight cytochrome c	Energy metabolism	Electron transport	

Predicted site	PromScan score	Start	End	Strand	Sequence
Promoter 63	83	3093435	3093451	+	AAGGCACGGAACATGCA

Operon/gene	Strand	Start	End	GSU	Gene	Annotation	Functional category	Functional subcategory	COG
1269 Tu 1	+	3093614	3095863	GSU2815		sensory box histidine kinase	Signal transduction	Two-component systems	## COG0642

Predicted site	PromScan score	Start	End	Strand	Sequence
Promoter 64	83	3027520	3027536	-	TTGGTATCCCCCTTGCT

Operon/gene	Strand	Start	End	GSU	Gene	Annotation	Functional category	Functional subcategory	COG
1232 Op 1	-	3025675	3026046	GSU2750		hypothetical protein	Hypothetical proteins	Domain	
1232 Op 2	-	3026124	3027449	GSU2751	dcuB	C4-dicarboxylate transporter, anaerobic	Transport and binding proteins	Carbohydrates, organic alcohols, and acids	## COG2704

Predicted site	PromScan score	Start	End	Strand	Sequence
Promoter 65	83	2765786	2765802	+	ATGGCACTCAACTTGAA

Operon/gene	Strand	Start	End	GSU	Gene	Annotation	Functional category	Functional subcategory	COG
1122 Tu 1	+	2765849	2767465	GSU2508		TPR domain protein	Unknown function	General	

Predicted site	PromScan score	Start	End	Strand	Sequence
Promoter 66	83	2129841	2129857	-	GTGGTACGTATTATGCT

Operon/gene	Strand	Start	End	GSU	Gene	Annotation	Functional category	Functional subcategory	COG
894 Tu 1	-	2128966	2129685	GSU1944		hypothetical protein	Not available	Not available	

Predicted site	PromScan score	Start	End	Strand	Sequence
Promoter 67	83	197880	197896	-	ACGGCACGACAACTGCT

Operon/gene	Strand	Start	End	GSU	Gene	Annotation	Functional category	Functional subcategory	COG
87 Op 1	-	196023	196325	GSU0182		lipoprotein, putative	Cell envelope	Other	
87 Op 2	-	196424	197731	GSU0183		lipoprotein, putative	Cell envelope	Other	## COG1376

Predicted site	PromScan score	Start	End	Strand	Sequence
Promoter 68	83	1397366	1397382	+	TTGGCATTGCAGCTGCT

Operon/gene	Strand	Start	End	GSU	Gene	Annotation	Functional category	Functional subcategory	COG
593 Tu 1	+	1397632	1398942	GSU1282		hypothetical protein	Not available	Not available	

Predicted site	PromScan score	Start	End	Strand	Sequence
Promoter 69	83	1039939	1039955	+	TTGGTATACAGGTTGCT

Operon/gene	Strand	Start	End	GSU	Gene	Annotation	Functional category	Functional subcategory	COG
449 Op 1	+	1040081	1040272	GSU0964		hypothetical protein	Not available	Not available	
449 Op 2	+	1040294	1040416						
449 Op 3	+	1040397	1040648	GSU0966		hypothetical protein	Not available	Not available	
449 Op 4	+	1040661	1041713	GSU0967		membrane protein, putative	Cell envelope	Other	

Predicted site	PromScan score	Start	End	Strand	Sequence
Promoter 70	82	860308	860324	-	CCGGCAAGATCATTGCA

Operon/gene	Strand	Start	End	GSU	Gene	Annotation	Functional category	Functional subcategory	COG
359 Tu 1	-	858649	860052	GSU0801		hypothetical protein	Not available	Not available	

Predicted site	PromScan score	Start	End	Strand	Sequence
Promoter 71	82	3072482	3072498	+	CTGGCAAGTGCCCTGCA

Operon/gene	Strand	Start	End	GSU	Gene	Annotation	Functional category	Functional subcategory	COG
1259 Tu 1	+	3072594	3073025	GSU2794	mscL	large conductance mechanosensitive channel protein	Cellular processes	Adaptations to atypical conditions	## COG1970

Predicted site	PromScan score	Start	End	Strand	Sequence
Promoter 72	82	3005233	3005249	+	AGGGCATCGATATTGCT

Operon/gene	Strand	Start	End	GSU	Gene	Annotation	Functional category	Functional subcategory	COG
1220 Tu 1	+	3005347	3005799	GSU2727		hypothetical protein	Not available	Not available	

Predicted site	PromScan score	Start	End	Strand	Sequence
Promoter 73	82	2798300	2798316	-	TTCGCCCGACTATTGCT

Operon/gene	Strand	Start	End	GSU	Gene	Annotation	Functional category	Functional subcategory	COG
1134 Op 1	-	2791476	2794637	GSU2531		sensory box histidine kinase	Signal transduction	Two-component systems	## COG0642
1134 Op 2	-	2794634	2795065	GSU2532		hypothetical protein	Hypothetical proteins	Conserved	
1134 Op 3	-	2795087	2796298	GSU2533		hypothetical protein	Not available	Not available	
1134 Op 4	-	2796313	2797671	GSU2534		sensory box/response regulator	Signal transduction	Two-component systems	## COG2199
1134 Op 5	-	2797751	2798254	GSU2535		response regulator	Signal transduction	Two-component systems	## COG0784

Predicted site	PromScan score	Start	End	Strand	Sequence
Promoter 74	82	1651315	1651331	+	GTGGCGCTTGAGTTGCA

Operon/gene	Strand	Start	End	GSU	Gene	Annotation	Functional category	Functional subcategory	COG
691 Tu 1	+	1651532	1652161	GSU1506		hypothetical protein	Not available	Not available	

Predicted site	PromScan score	Start	End	Strand	Sequence
Promoter 75	82	1455058	1455074	+	ATGGCACGGCCTGTGTA

Operon/gene	Strand	Start	End	GSU	Gene	Annotation	Functional category	Functional subcategory	COG
614 Op 1	+	1455116	1456396	GSU1330		metal ion efflux outer membrane protein family protein, putative	Transport and binding proteins	Cations and iron carrying compounds	## COG1538
614 Op 2	+	1456441	1457775	GSU1331		efflux transporter, RND family, MFP subunit	Transport and binding proteins	Unknown substrate	## COG0845
614 Op 3	+	1457786	1460923	GSU1332		heavy metal efflux pump, CzcA family	Transport and binding proteins	Cations and iron carrying compounds	## COG3696
614 Op 4	+	1460959	1461432	GSU1333		hypothetical protein	Not available	Not available	

Predicted site	PromScan score	Start	End	Strand	Sequence
Promoter 76	81	773999	774015	+	TTGGCACGGGACATGAA

Operon/gene	Strand	Start	End	GSU	Gene	Annotation	Functional category	Functional subcategory	COG
313 Op 1	+	774053	774286	GSU0725		hypothetical protein	Not available	Not available	
313 Op 2	+	774344	774949	GSU0726		chemotaxis protein CheD, putative	Cellular processes	Chemotaxis and motility	## COG1871

Predicted site	PromScan score	Start	End	Strand	Sequence
Promoter 77	81	744982	744998	+	TTGGTATTCAATTTGCT

Operon/gene	Strand	Start	End	GSU	Gene	Annotation	Functional category	Functional subcategory	COG
306 Op 1	+	745248	750929	GSU0702		cytochrome c family protein	Energy metabolism	Electron transport	
306 Op 2	+	751019	751543	GSU0703		hypothetical protein	Not available	Not available	
306 Op 3	+	751546	752874	GSU0704		hypothetical protein	Not available	Not available	
306 Op 4	+	752897	753697	GSU0705		cytochrome c biogenesis protein, CcmF/CcyK/CcsA family	Energy metabolism	Electron transport	## COG0755

Predicted site	PromScan score	Start	End	Strand	Sequence
Promoter 78	81	628383	628399	-	CTGGCATACGGGGTGCA

Operon/gene	Strand	Start	End	GSU	Gene	Annotation	Functional category	Functional subcategory	COG
262 Op 1	-	625543	626199	GSU0593		hypothetical protein	#N/A	#N/A	
262 Op 2	-	626203	627252	GSU0594		cytochrome c family protein	Energy metabolism	Electron transport	
262 Op 3	-	627343	627576	GSU0595.1		hypothetical protein	Not available	Not available	
262 Op 4	-	627650	628042	GSU0596		response regulator	Signal transduction	Two-component systems	
262 Op 5	-	628093	628335	GSU0597		hypothetical protein	Not available	Not available	

Predicted site	PromScan score	Start	End	Strand	Sequence
Promoter 79	81	555596	555612	-	TTGACACGCTTTGTGCT

Operon/gene	Strand	Start	End	GSU	Gene	Annotation	Functional category	Functional subcategory	COG
226 Op 1	-	549749	550540	GSU0516		hypothetical protein	Hypothetical proteins	Conserved	## COG1082
226 Op 2	-	550546	551760	GSU0517		hypothetical protein	#N/A	#N/A	## COG2905
226 Op 3	-	551782	553305	GSU0518		sodium/solute symporter family protein	Transport and binding proteins	Unknown substrate	## COG0591
226 Op 4	-	553298	553624	GSU0519		hypothetical protein	Hypothetical proteins	Conserved	
226 Op 5	-	553701	554927	GSU0520		hypothetical protein	Hypothetical proteins	Conserved	## COG0585
226 Op 6	-	554931	555560	GSU0521		methyltransferase, putative	Unknown function	Enzymes of unknown specificity	## COG0220

Predicted site	PromScan score	Start	End	Strand	Sequence
Promoter 80	81	465979	465995	-	CGGGCACCGCGGTTGCC

Operon/gene	Strand	Start	End	GSU	Gene	Annotation	Functional category	Functional subcategory	COG
192 Tu 1	-	464944	465948	GSU0434		hypothetical protein	Hypothetical proteins	Conserved	## COG0482

Predicted site	PromScan score	Start	End	Strand	Sequence
Promoter 81	81	3546239	3546255	-	ACGGCAGGTAAATTGCT

Operon/gene	Strand	Start	End	GSU	Gene	Annotation	Functional category	Functional subcategory	COG
1412 Op 1	-	3543991	3544680	GSU3229		DNA-binding response regulator, LuxR family	Signal transduction	Two-component systems	## COG2197
1412 Op 2	-	3544670	3546151	GSU3230		sensory box histidine kinase	Signal transduction	Two-component systems	## COG0642

Predicted site	PromScan score	Start	End	Strand	Sequence
Promoter 82	81	3503117	3503133	+	ACGGAACACTTCTTGCT

Operon/gene	Strand	Start	End	GSU	Gene	Annotation	Functional category	Functional subcategory	COG
1400 Op 1	+	3503177	3504052	GSU3195		chemotaxis protein methyltransferase CheR,putative	Cellular processes	Chemotaxis and motility	## COG1352
1400 Op 2	+	3504112	3505398	GSU3196		methyl-accepting chemotaxis protein	Cellular processes	Chemotaxis and motility	## COG0840
1400 Op 3	+	3505407	3505811	GSU3197		purine-binding chemotaxis protein CheW, putative	Cellular processes	Chemotaxis and motility	
1400 Op 4	+	3505849	3506193	GSU3198	cheY-7	chemotaxis protein CheY	Cellular processes	Chemotaxis and motility	## COG0784
1400 Op 5	+	3506196	3507881	GSU3199	cheA-3	chemotaxis protein CheA	Cellular processes	Chemotaxis and motility	## COG0643
1400 Op 6	+	3507878	3508492	GSU3200		chemotaxis protein, CheC family	Cellular processes	Chemotaxis and motility	## COG1776
1400 Op 7	+	3508489	3508977	GSU3201		chemotaxis protein CheD, putative	Cellular processes	Chemotaxis and motility	## COG1871
1400 Op 8	+	3509017	3509829	GSU3202		hypothetical protein	Not available	Not available	

Predicted site	PromScan score	Start	End	Strand	Sequence
Promoter 83	81	3179817	3179833	-	CTGGTATATTCTTTGCT

Operon/gene	Strand	Start	End	GSU	Gene	Annotation	Functional category	Functional subcategory	COG
1298 Op 1	-	3178124	3178315	GSU2892		hypothetical protein	Not available	Not available	
1298 Op 2	-	3178281	3179501	GSU2893		hypothetical protein	Not available	Not available	

Predicted site	PromScan score	Start	End	Strand	Sequence
Promoter 84	81	3105888	3105904	-	TTGGAACACTTTATGCT

Operon/gene	Strand	Start	End	GSU	Gene	Annotation	Functional category	Functional subcategory	COG
1275 Tu 1	-	3105261	3105785	GSU2822		response regulator	Signal transduction	Two-component systems	## COG3707

Predicted site	PromScan score	Start	End	Strand	Sequence
Promoter 85	81	3079447	3079463	-	GTGGCACGCTTAGTGTT

Operon/gene	Strand	Start	End	GSU	Gene	Annotation	Functional category	Functional subcategory	COG
1261 Op 1	-	3077578	3078381	GSU2798		acetyltransferase, GNAT family	Unknown function	Enzymes of unknown specificity	## COG0454
1261 Op 2	-	3078386	3079258	GSU2799		radical SAM domain protein	Unknown function	Enzymes of unknown specificity	## COG0535

Predicted site	PromScan score	Start	End	Strand	Sequence
Promoter 86	81	2842541	2842557	-	TTGGCATCCTGCCTGCT

Operon/gene	Strand	Start	End	GSU	Gene	Annotation	Functional category	Functional subcategory	COG
1149 Op 1	-	2834710	2835063	GSU2573	cysE	serine acetyltransferase	Amino acid biosynthesis	Serine family	
1149 Op 2	-	2835091	2836197	GSU2574		response regulator	Signal transduction	Two-component systems	## COG3437
1149 Op 3	-	2836201	2838753	GSU2575		sensor histidine kinase/response regulator	Signal transduction	Two-component systems	## COG0642
1149 Op 4	-	2838754	2839617	GSU2576		HD domain protein	Unknown function	General	## COG1639
1149 Op 5	-	2839604	2839939	GSU2577		hypothetical protein	Not available	Not available	
1149 Op 6	-	2840026	2840532	GSU2578	cheW-9	purine-binding chemotaxis protein CheW	Cellular processes	Chemotaxis and motility	## COG0835
1149 Op 7	-	2840564	2842195	GSU2579		methyl-accepting chemotaxis protein	Cellular processes	Chemotaxis and motility	## COG0840
1149 Op 8	-	2842215	2842481	GSU2580		hypothetical protein	Not available	Not available	

Predicted site	PromScan score	Start	End	Strand	Sequence
Promoter 87	81	2784628	2784644	-	TTGGCATGTAAATTGTA

Operon/gene	Strand	Start	End	GSU	Gene	Annotation	Functional category	Functional subcategory	COG
1130 Op 1	-	2783251	2783754	GSU2525		nitroreductase family protein	Unknown function	Enzymes of unknown specificity	## COG0778
1130 Op 2	-	2783754	2784488	GSU2526		membrane protein, putative	Cell envelope	Other	

Predicted site	PromScan score	Start	End	Strand	Sequence
Promoter 88	81	2597007	2597023	-	CTGGCACATATCTTACG

Operon/gene	Strand	Start	End	GSU	Gene	Annotation	Functional category	Functional subcategory	COG
1053 Op 1	-	2591302	2591688	GSU2364		mannose-6-phosphate isomerase	Energy metabolism	Sugars	## COG0662
1053 Op 2	-	2591704	2592546	GSU2365	rfbD	dTDP-4-dehydrorhamnose reductase	Cell envelope	Biosynthesis and degradation of surface polysacchar	## COG1091
1053 Op 3	-	2592543	2593619	GSU2366	rfbB	dTDP-glucose 4,6-dehydratase	Cell envelope	Biosynthesis and degradation of surface polysacchar	## COG1088
1053 Op 4	-	2593636	2595708	GSU2367		organic solvent tolerance protein, putative	Cellular processes	Detoxification	## COG1452
1053 Op 5	-	2595766	2596911	GSU2368	folC	folC bifunctional protein	Biosynthesis of cofactors, prostheti	Folic acid	## COG0285

Predicted site	PromScan score	Start	End	Strand	Sequence
Promoter 89	81	1651206	1651222	+	CTGGCATCGTCACTGCA

Operon/gene	Strand	Start	End	GSU	Gene	Annotation	Functional category	Functional subcategory	COG
691 Tu 1	+	1651532	1652161	GSU1506		hypothetical protein	Not available	Not available	

Predicted site	PromScan score	Start	End	Strand	Sequence
Promoter 90	81	1523169	1523185	-	GTGGCAAAGGCGCTGCA

Operon/gene	Strand	Start	End	GSU	Gene	Annotation	Functional category	Functional subcategory	COG
647 Tu 1	-	1522574	1523083						

Predicted site	PromScan score	Start	End	Strand	Sequence
Promoter 91	81	1353732	1353748	+	CTGGCATTGTTTTTGAT

Operon/gene	Strand	Start	End	GSU	Gene	Annotation	Functional category	Functional subcategory	COG
583 Op 1	+	1353842	1360090	GSU1251		BNR repeat domain protein	Unknown function	General	
583 Op 2	+	1360117	1365075	GSU1252		hypothetical protein	#N/A	#N/A	
583 Op 3	+	1365114	1367153	GSU1253		hypothetical protein	Not available	Not available	
583 Op 4	+	1367157	1368500	GSU1254		hypothetical protein	Not available	Not available	
583 Op 5	+	1368521	1369105	GSU1255		hypothetical protein	Hypothetical proteins	Conserved	## COG1999
583 Op 6	+	1369123	1369746	GSU1256		hypothetical protein	Hypothetical proteins	Domain	
583 Op 7	+	1369743	1371368	GSU1257		ABC transporter, periplasmic-substrate binding protein, putative	Transport and binding proteins	Unknown substrate	

Predicted site	PromScan score	Start	End	Strand	Sequence
Promoter 92	81	1329103	1329119	+	TTGGCTTCAGTATTGCA

Operon/gene	Strand	Start	End	GSU	Gene	Annotation	Functional category	Functional subcategory	COG
572 Tu 1	+	1329237	1329683	GSU1225		hypothetical protein	Not available	Not available	

Predicted site	PromScan score	Start	End	Strand	Sequence
Promoter 93	81	1121057	1121073	-	GCGGCACGAGATCTGCA

Operon/gene	Strand	Start	End	GSU	Gene	Annotation	Functional category	Functional subcategory	COG
480 Tu 1	-	1119359	1121023	GSU1036		sensor histidine kinase	Signal transduction	Two-component systems	## COG0642

Predicted site	PromScan score	Start	End	Strand	Sequence
Promoter 94	80	87167	87183	-	ATGGCACGTTTTATGAA

Operon/gene	Strand	Start	End	GSU	Gene	Annotation	Functional category	Functional subcategory	COG
35 Tu 1	-	86581	87030	GSU0071		hypothetical protein	Not available	Not available	

Predicted site	PromScan score	Start	End	Strand	Sequence
Promoter 95	80	854282	854298	-	ATGCCTCGCCGCTTGCA

Operon/gene	Strand	Start	End	GSU	Gene	Annotation	Functional category	Functional subcategory	COG
357 Op 1	-	852149	853831	GSU0794		pyridine nucleotide-disulfide oxidoreductase/rhodanese domain protein	Unknown function	General	## COG0446
357 Op 2	-	853894	854202	GSU0795		rhodanese-like domain protein	Unknown function	General	## COG0607

Predicted site	PromScan score	Start	End	Strand	Sequence
Promoter 96	80	849091	849107	-	CTGGCATTATTTTTGTT

Operon/gene	Strand	Start	End	GSU	Gene	Annotation	Functional category	Functional subcategory	COG
354 Op 1	-	848545	848949	GSU0789		response regulator	Signal transduction	Two-component systems	## COG2204
354 Op 2	-	849136	850056	GSU0790		hypothetical protein	Not available	Not available	

Predicted site	PromScan score	Start	End	Strand	Sequence
Promoter 97	80	716820	716836	+	TTGGCACGAATTTTTTT

Operon/gene	Strand	Start	End	GSU	Gene	Annotation	Functional category	Functional subcategory	COG
294 Op 1	+	716939	718009	GSU0681		sensory box histidine kinase	Signal transduction	Two-component systems	## COG0642
294 Op 2	+	718009	718656	GSU0682		DNA-binding response regulator, LuxR family	Signal transduction	Two-component systems	## COG2197

Predicted site	PromScan score	Start	End	Strand	Sequence
Promoter 98	80	3462928	3462944	+	ACGGCACCGGCATTGCC

Operon/gene	Strand	Start	End	GSU	Gene	Annotation	Functional category	Functional subcategory	COG
1383 Tu 1	+	3463053	3464168	GSU3156		methyl-accepting chemotaxis protein, putative	Cellular processes	Chemotaxis and motility	## COG0840

Predicted site	PromScan score	Start	End	Strand	Sequence
Promoter 99	80	2699909	2699925	+	ACGGGACGGGGATTGCA

Operon/gene	Strand	Start	End	GSU	Gene	Annotation	Functional category	Functional subcategory	COG
1096 Tu 1	+	2700316	2701422	GSU2462		homoserine O-acetyltransferase	Amino acid biosynthesis	Aspartate family	## COG2021

Predicted site	PromScan score	Start	End	Strand	Sequence
Promoter 100	80	2247542	2247558	-	AAGGCCTGAATCTTGCT

Operon/gene	Strand	Start	End	GSU	Gene	Annotation	Functional category	Functional subcategory	COG
926 Tu 1	-	2246279	2247466	GSU2047		hypothetical protein	Hypothetical proteins	Conserved	## COG1680

Predicted site	PromScan score	Start	End	Strand	Sequence
Promoter 101	80	2186329	2186345	+	TGGGCACAACAAATGCT

Operon/gene	Strand	Start	End	GSU	Gene	Annotation	Functional category	Functional subcategory	COG
908 Tu 1	+	2186413	2187024	GSU1994		hypothetical protein	Not available	Not available	

Predicted site	PromScan score	Start	End	Strand	Sequence
Promoter 102	80	2135268	2135284	-	ATGGCAAATATTTAGCT

Operon/gene	Strand	Start	End	GSU	Gene	Annotation	Functional category	Functional subcategory	COG
896 Op 1	-	2133874	2134860						
896 Op 2	-	2134875	2135165	GSU1948		hypothetical protein	Not available	Not available	

Predicted site	PromScan score	Start	End	Strand	Sequence
Promoter 103	80	2002713	2002729	-	TAGGCAGACTATTTGCT

Operon/gene	Strand	Start	End	GSU	Gene	Annotation	Functional category	Functional subcategory	COG
843 Op 1	-	2000819	2002231	GSU1835	glnA	glutamine synthetase, type I	Amino acid biosynthesis	Glutamate family	## COG0174
843 Op 2	-	2002270	2002608	GSU1836		nitrogen regulatory protein P-II	Regulatory functions	Protein interactions	## COG0347

Predicted site	PromScan score	Start	End	Strand	Sequence				
Promoter 104	80	191151	191167	+	GTGGCACCGGGTTGGCG				
**Operon/gene**	**Strand**	**Start**	**End**	**GSU**	**Gene**	**Annotation**	**Functional category**	**Functional subcategory**	**COG**
81 Tu 1	+	191349	191960	GSU0175		transcriptional regulator, TetR family	Regulatory functions	DNA interactions	## COG1309

Predicted site	PromScan score	Start	End	Strand	Sequence				
Promoter 105	80	1874885	1874901	+	AGGGCAAGCTACTTGCG				
**Operon/gene**	**Strand**	**Start**	**End**	**GSU**	**Gene**	**Annotation**	**Functional category**	**Functional subcategory**	**COG**
792 Tu 1	+	1874995	1876245	GSU1709.1	smpB	SsrA-binding protein	Protein synthesis	Other	## COG0582

Predicted site	PromScan score	Start	End	Strand	Sequence
Promoter 106	80	1843205	1843221	+	AAGGCACTGTTCCTGCA

Operon/gene	Strand	Start	End	GSU	Gene	Annotation	Functional category	Functional subcategory	COG
782 Op 1	+	1843279	1843620	GSU1682		lipoprotein, putative	Cell envelope	Other	
782 Op 2	+	1843617	1844066	GSU1683		hypothetical protein	Hypothetical proteins	Conserved	

Predicted site	PromScan score	Start	End	Strand	Sequence
Promoter 107	80	181724	181740	+	TTGGAATTCGAGTTGCT

Operon/gene	Strand	Start	End	GSU	Gene	Annotation	Functional category	Functional subcategory	COG
74 Tu 1	+	182339	182458						

Predicted site	PromScan score	Start	End	Strand	Sequence
Promoter 108	80	1540183	1540199	+	GTCGCCCGCACGTTGCT

Operon/gene	Strand	Start	End	GSU	Gene	Annotation	Functional category	Functional subcategory	COG
657 Op 1	+	1540372	1543839	GSU1401	dnaE	DNA polymerase III, alpha subunit	DNA metabolism	DNA replication, recombination, and repair	## COG0587
657 Op 2	+	1543877	1544836	GSU1402	accA	acetyl-CoA carboxylase, carboxyl transferase, alpha subunit	Fatty acid and phospholipid metab	Biosynthesis	## COG0825

Predicted site	PromScan score	Start	End	Strand	Sequence
Promoter 109	80	1380900	1380916	-	TCGGCACGCATTTTGTA

Operon/gene	Strand	Start	End	GSU	Gene	Annotation	Functional category	Functional subcategory	COG
587 Tu 1	-	1379856	1380863	GSU1268		transcriptional regulator, LysR family	Regulatory functions	DNA interactions	## COG0583

Predicted site	PromScan score	Start	End	Strand	Sequence
Promoter 110	80	1293612	1293628	-	ATGGCATGCAAATGGCC

Operon/gene	Strand	Start	End	GSU	Gene	Annotation	Functional category	Functional subcategory	COG
553 Tu 1	-	1292703	1293191	GSU1185		hypothetical protein	Hypothetical proteins	Conserved	## COG1839
