# Supplementary material for: Genome-wide analysis of the RpoN regulon in Geobacter sulfurreducens
Source: BMC Genomics. 2009 Jul 22;10:331. doi: 10.1186/1471-2164-10-331 (PMC2725144; doi:10.1186/1471-2164-10-331)
Supplement: Additional file 5 — List of operons/genes containing RpoN-dependent promoters identified by the PromScan analysis and also identified using transcriptome analysis. [file 1471-2164-10-331-S5.pdf]

Additional file 5. List of operons/genes containing RpoN-dependent promoters identified by the PromScan analysis and also identified using transcriptome analysis.

1. Promoter # are based on the PromScan scores and are the same as those reported in additional files 3 & 4.
2. The search is based on fold change cutoff at 1.25 and P<0.0005.
3. N.D.: Not detected. The fold changes of the gene did not match the differential expression criteria.

Promoter #	GSU	Gene	Annotation	Fold changes
**1**	GSU3050		flagella basal body P-ring formation protein flgA, putative	N.D.
	GSU3051	*flgG-1*	flagellar basal-body rod protein FlgG	N.D.
	GSU3052	*flgG-2*	flagellar basal-body rod protein FlgG	N.D.
	GSU3053	*fliA*	RNA polymerase sigma factor for flagellar Operon/gene/gene	N.D.
	GSU3054		ParA family protein	N.D.
	GSU3055		flagellar biosynthetic protein FlhF, putative	-1.34
	GSU3056	*flhA*	flagellar biosynthetic protein FlhA	N.D.
**2**	GSU0937	*nifV*	homocitrate synthase	-1.47
**3**	GSU0407	*flgB*	flagellar basal-body rod protein FlgB	N.D.
	GSU0408	*flgC*	flagellar basal-body rod protein FlgC	-1.31
**5**	GSU2005		branched-chain amino acid ABC transporter, periplasmic amino acid-bindi	1.38
	GSU2006		branched-chain amino acid ABC transporter, permease protein	1.29
	GSU2007		branched-chain amino acid ABC transporter, permease protein	N.D.
	GSU2008		branched-chain amino acid ABC transporter, ATP-binding protein	N.D.
	GSU2009		branched-chain amino acid ABC transporter, ATP-binding protein	1.23
	GSU2010		CBS domain protein	N.D.
**10**	GSU0938		hypothetical protein	N.D.
	GSU0939		nitrogen regulatory protein P-II, putative	-1.40
	GSU0940		ammonium transporter	N.D.
**11**	GSU0777	*fdnG*	formate dehydrogenase, major subunit, selenocysteine-containing	-1.20
	GSU0777	*fdnG*	formate dehydrogenase, major subunit, selenocysteine-containing	-1.20
	GSU0778	*fdnH*	formate dehydrogenase, iron-sulfur subunit	N.D.

	GSU0779	formate dehydrogenase, b-type cytochrome subunit, putative	N.D.
	GSU0780	formate dehydrogenase accessory protein FdhD	N.D.
	GSU0781	twin-arginine translocation protein, TatA/E family	-1.41
**12**	GSU3409	hypothetical protein	N.D.
	GSU3410	hypothetical protein	3.31
**13**	GSU2938	hypothetical protein	-1.49
	GSU2939	outer membrane porin FmdC, putative	N.D.
	GSU2940	lipoprotein, putative	N.D.
**18**	GSU2634	hypothetical protein	N.D.
	GSU2635	hypothetical protein	N.D.
	GSU2636	alpha-amylase family protein	1.16
	GSU2637	alcohol dehydrogenase, zinc-containing	1.31
**19**	GSU2202	transcriptional regulator, LysR family	-1.30
**20**	GSU1235	hypothetical protein	N.D.
	GSU1236	hypothetical protein	N.D.
	GSU1237	pyridine nucleotide-disulphide oxidoreductase family protein	-1.44
	GSU1238	iron-sulfur cluster-binding protein	N.D.
	GSU1239	glutamate synthase-related protein	N.D.
**25**	GSU0420	flagellar protein FliL	-1.47
	GSU0421 *fliM*	flagellar motor switch protein FliM	N.D.
	GSU0422 *fliN*	flagellar motor switch protein FliN	-1.93
	GSU0422.1*fliN*	flagellar motor switch protein FliN	N.D.
	GSU0423 *fliP*	flagellar biosynthetic protein FliP	N.D.
	GSU0424 *fliQ*	flagellar biosynthetic protein FliQ	N.D.
	GSU0425 *fliR*	flagellar biosynthesis protein FliR	-1.72
	GSU0426 *flhB*	flagellar biosynthetic protein FlhB	N.D.

**27**	GSU3175		hypothetical protein	N.D.
	GSU3176		lysM domain protein	-1.25
	GSU3177		Rhs family protein	N.D.
	GSU3178		hypothetical protein	-1.31
	GSU3179		hypothetical protein	N.D.
**28**	GSU3040		hypothetical protein	N.D.
	GSU3041	*csrA*	carbon storage regulator	N.D.
	GSU3042	*flgL*	flagellar hook-associated protein FlgL	N.D.
	GSU3043	*flgK*	flagellar hook-associated protein FlgK	N.D.
	GSU3044		hypothetical protein	N.D.
	GSU3045	*flgM*	negative regulator of flagellin synthesis FlgM	N.D.
	GSU3046	*flgJ*	flagellar protein FlgJ-like protein	-1.41
**29**	GSU3017		hypothetical protein	N.D.
	GSU3018		hypothetical protein	1.44
	GSU3019		dehydrogenase, E1 component, alpha and beta subunits	N.D.
	GSU3020		hexapeptide transferase family protein	N.D.
	GSU3021		DegT/DnrJ/EryC1/StrS family protein	N.D.
	GSU3022		hypothetical protein	N.D.
	GSU3023		glycosyl transferase, group 38354 family protein	N.D.
	GSU3024		hypothetical protein	1.15
	GSU3025		heptosyltransferase family protein	N.D.
	GSU3026		flagellar protein FlbD, putative	N.D.
	GSU3027		chemotaxis MotA protein	N.D.
	GSU3028	*motB*	chemotaxis MotB protein	N.D.
**31**	GSU2802	*dRAT*	NAD(+)--dinitrogen-reductase ADP-D-ribosyltransferase	N.D.
	GSU2803		dinitrogenase iron-molybdenum cofactor family protein	N.D.
	GSU2804		ferredoxin family protein	-1.24
	GSU2805	*nifX*	nitrogenase molybdenum-iron cofactor biosynthesis protein NifX	N.D.
	GSU2806	*nifEN*	nitrogenase molybdenum-iron cofactor biosynthesis protein NifEN	-1.27

**32**	GSU2622		HAMP domain/GAF domain/HD domain protein	-1.35
**34**	GSU2476		TPR domain protein	N.D.
	GSU2477		TPR domain/radical SAM/B12 binding domain protein	-1.26
**35**	GSU0098		MglB protein	N.D.
	GSU0099		MglA protein	1.43
**40**	GSU2908		hypothetical protein	-1.34
	GSU2909		lipoprotein, putative	N.D.
	GSU2910		hypothetical protein	N.D.
	GSU2911		hypothetical protein	-1.33
**42**	GSU2490		oxalate/formate antiporter, putative	1.36
**44**	GSU2034		hypothetical protein	-1.32
	GSU2035		hypothetical protein	-1.33
	GSU2036		hypothetical protein	-1.66
	GSU2037		hypothetical protein	N.D.
	GSU2038		hypothetical protein	N.D.
	GSU2039		hypothetical protein	N.D.
**47**	GSU0618	*omcE*	cytochrome c family protein	1.26
**48**	GSU0357		cytochrome c family protein	1.24
	GSU0358		iron-sulfur cluster-binding protein	N.D.
**50**	GSU2884		cytochrome c family protein	-1.34
	GSU2885		NHL repeat domain protein	N.D.
**51**	GSU2378	*trpF*	N-(5'phosphoribosyl)anthranilate isomerase	-1.48
	GSU2379		pyridoxal-phosphate dependent enzyme	N.D.
	GSU2380	*trpC*	Indole-3-glycerol phosphate synthase	N.D.

	GSU2381	*trpD*	anthranilate phosphoribosyltransferase	N.D.
	GSU2382	*trpG*	anthranilate synthase component II	N.D.
	GSU2383	*trpE*	anthranilate synthase component I	N.D.
	GSU2384		sensor histidine kinase	N.D.
	GSU2385		hypothetical protein	-1.30
	GSU2386		methylcobamide:CoM methyltransferase-related protein	N.D.
	GSU2387		B12-binding domain	N.D.
	GSU2388		sensory box histidine kinase	-1.27
	GSU2389		ABC transporter, periplasmic substrate-binding protein, putative	N.D.
**58**	GSU0930		sulfur transferase, putative, selenocysteine-containing	-1.34
**60**	GSU3364		hypothetical protein	1.45
**61**	GSU3206		dnaK suppressor protein, putative	1.56
**63**	GSU2815		sensory box histidine kinase	1.22
**64**	GSU2750		hypothetical protein	1.30
	GSU2751	*dcuB*	C4-dicarboxylate transporter, anaerobic	1.63
**66**	GSU1944		hypothetical protein	-1.38
**73**	GSU2531		sensory box histidine kinase	N.D.
	GSU2532		hypothetical protein	N.D.
	GSU2533		hypothetical protein	N.D.
	GSU2534		sensory box/response regulator	-1.26
	GSU2535		response regulator	-1.28
**77**	GSU0702		cytochrome c family protein	N.D.
	GSU0703		hypothetical protein	N.D.
	GSU0704		hypothetical protein	N.D.
	GSU0705		cytochrome c biogenesis protein, CcmF/CcyK/CcsA family	-1.28

**79**	GSU0516		hypothetical protein	N.D.
	GSU0517		hypothetical protein	N.D.
	GSU0518		sodium/solute symporter family protein	-1.44
	GSU0519		hypothetical protein	N.D.
	GSU0520		hypothetical protein	N.D.
	GSU0521		methyltransferase, putative	N.D.
**81**	GSU3229		DNA-binding response regulator, LuxR family	-1.36
	GSU3230		sensory box histidine kinase	N.D.
**82**	GSU3195		chemotaxis protein methyltransferase CheR,putative	-1.27
	GSU3196		methyl-accepting chemotaxis protein	N.D.
	GSU3197		purine-binding chemotaxis protein CheW, putative	N.D.
	GSU3198	*cheY-7*	chemotaxis protein CheY	N.D.
	GSU3199	*cheA-3*	chemotaxis protein CheA	N.D.
	GSU3200		chemotaxis protein, CheC family	N.D.
	GSU3201		chemotaxis protein CheD, putative	N.D.
	GSU3202		hypothetical protein	N.D.
**87**	GSU2525		nitroreductase family protein	-1.53
	GSU2526		membrane protein, putative	N.D.
**91**	GSU1251		BNR repeat domain protein	N.D.
	GSU1252		hypothetical protein	N.D.
	GSU1253		hypothetical protein	-1.41
	GSU1254		hypothetical protein	N.D.
	GSU1255		hypothetical protein	N.D.
	GSU1256		hypothetical protein	N.D.
	GSU1257		ABC transporter, periplasmic-substrate binding protein, putative	N.D.
**101**	GSU1994		hypothetical protein	1.90

**103**	GSU1835	*glnA*	glutamine synthetase, type I	N.D.
	GSU1836	*glnB*	nitrogen regulatory protein PII	1.77
**108**	GSU1401	*dnaE*	DNA polymerase III, alpha subunit	-1.37
	GSU1402	*accA*	acetyl-CoA carboxylase, carboxyl transferase, alpha subunit	1.77
