# Supplementary material for: Genome-wide analysis of the RpoN regulon in Geobacter sulfurreducens
Source: BMC Genomics. 2009 Jul 22;10:331. doi: 10.1186/1471-2164-10-331 (PMC2725144; doi:10.1186/1471-2164-10-331)
Supplement: Additional file 6 — RpoN-dependent gene expression. Representative genes, (a) GSU0364, GSU2005, GSU2302, GSU2490 and GSU3206 (up-regulated in the RpoN+ strain), and (b) GSU0938 (down-regulated in the RpoN+ strain) identified by the microarray analysis were analyzed by primer extension assays. The results of the primer extension assays and their promoter regions are shown. The 5' ends of mRNA are indicated by asterisks. RBS sites are underlined. Translation start codons are in bold and are indicated by Met. [file 1471-2164-10-331-S6.pdf]

a

A G C T WT^{V} RpoN^{+}

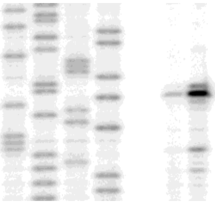

GSU0364-PpcB

ATAGATGTCGATGGCGGACAGGGCGCTGGCCTCC
-35
TACACTGGGACC**A**TGCGATTTCGCACACACCCCCG
-10
AAAGGAGAATCACC**ATG**
RBS **Met**

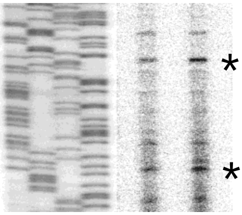

GSU2005-ABC Transporter

AAAGCCGGTCAGAGAGCAGATTTGCCCCAGTTAT
ACCCGAACT**G**GGCGTTCTGGAAATGTATTTTTGA
AGTGTTATTATAAAGTATTGTCAGTCAC**T**ACCCAC
CAAGGAGGCAGAAC**ATG**
RBS **Met**

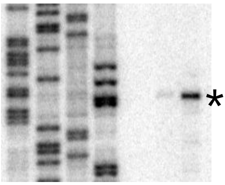

GSU2302-Trehalose phosphatase

CCCTGTCAACCGATAGTGCGCTCGGCCGTTCTTT
-35 -10
ATCAT**T**AATTTCGGCCGCAACAGCATTCCGGGAA
ACCGTCCGCCGCGGTCCCGGGACAACGGAGGTG
RBS
CAACG**ATG**
Met

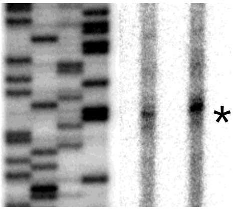

GSU2490-oxalate/formate antiporter

GCCGTTATAACATTATGTTTTTCGTTACTTTTTA
AAATAAACATAATTTTATAACAATGGTATGCA**A**G
TTGCTCTACCTCAAACAAACGCACTTCGTTCGTC
CACAGAGTCTGAACTCGTTCATAAGGAGGTAGCG
RBS
CG**ATG**
Met

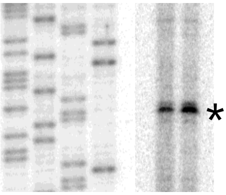

GSU3206-DksA

TTGCTTGACTTCAAAAAAGTTTTCGGTATCATTTC
GT**G**GCTCTTGAGGCCGGAAAATCAGCAGAGGGA
RBS
GATAGTAA**ATG**
Met

b

A G C T WT^{V} RpoN^{+}

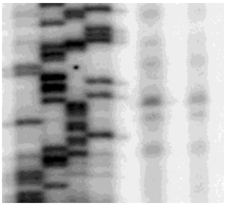

GSU0939-Nitrogen regulatory protein

GCACAACGCTGTGCCATCCATGCAACGAAATCGG
GTAACCAGGCAAAGGCGCCTTCCACCAC**G**GGTGG
AGGCGCCTTTCTTTTTTTACATTGTGCGGAAAGGA
GGAGAAGCTGAACGTCGTTGGGAGGACATTCGTG
AATGCAATTATTACTGACAAAGGAGAAGCATCA
RBS
ATG
Met
