# Supplementary material for: Genome-wide analysis of the RpoN regulon in Geobacter sulfurreducens
Source: BMC Genomics. 2009 Jul 22;10:331. doi: 10.1186/1471-2164-10-331 (PMC2725144; doi:10.1186/1471-2164-10-331)
Supplement: Additional file 7 — Primers used in this work. [file 1471-2164-10-331-S7.pdf]

Additional file 7. Primers used in this work

Primers	Sequences (5'-3')	Usage
rpoNU-1	CGGCGAAGAGGACATCACG	Mutagenesis
rpoNU-2	AGAGTCTGAATGATTCTCCC	Mutagenesis
rpoNU-3	GGGAGAATCATTCAGACTCTACCTGGGATGAATGTCAGCTAC	Mutagenesis
rpoNU-4	CCGGCTGCAGAACCGGCTGGCAGAAGGCGGCGGTGGAATCG	Mutagenesis
rpoNU-5	GCCAGCCGGTTCTGCAGCCGG	Mutagenesis
rpoNU-6	GCCGTCTTCATCTATGTTGC	Mutagenesis
rpoND-1	CCCAATCTGAGAATCAG	Mutagenesis
rpoND-2	CAAAATGTCAGCTGGTC	Mutagenesis
rpoND-3	GACCAGCTGACATTTTGACCTGGGATGAATGTCAGCTAC	Mutagenesis
rpoND-4	CAAAGCACGAAATGACGAGAAGGCGGCGGTGGAATCG	Mutagenesis
rpoND-5	CGTCATTTCGTGCTTTG	Mutagenesis
rpoND-6	TCCTTGTGCAGAAGATCC	Mutagenesis
Cm-rpoNF1	GGCGCCACGGAAGATCACTTCGC	Mutagenesis
Cm-rpoNR2	AGAGTCTGAATGATTCTCCCAGGGCACCAATAACTGCC	Mutagenesis
C-rpoNF3	GGGAGAATCATTCAGACTCT	Mutagenesis
C-rpoNR4	GGCGCCCAAAGCACGAAATGACGATC	Mutagenesis
ppcAF1	CGTTCCCGTCATGGAAGAAG	Mutagenesis
ppcAR2	GGAACAGAAGAAATCCCACAG	Mutagenesis
RpoNKmII-1	CTGGTCCGTCAGGAGATGG	Mutagenesis
RpoNKmII-2	GGATCAGGAGACATTCCCTC	Mutagenesis
RpoNKmII-5	CACCTCCACGACTTGGAG	Mutagenesis
RpoNKmII-6	TACGTCCCTGAGCACCAG	Mutagenesis
RpoNKmII-3	GAGGGAATGTCTCCTGATCCACCTGGGATGAATGTCAGCTAC	Mutagenesis
RpoNKmII-4	CTCCAAGTCGTGGAGGTGAGAAGGCGGCGGTGGAATCG	Mutagenesis
RpoNfor-XbaI	GGTCTAGACGGATCTTCCTTCAAAGGTG	RpoN expression
RpoNrev-EcoRI	GGGAATTCGTCAGCTGGTCAAAAATGAC	RpoN expression
RpoNfor-EcoRI	GG*GAATTC*GGATCTTCCTTCAAAGGTG	RpoN overexpression

RpoNrev-HindIII	GGAAGCTTGTCAGCTGGTCAAAAATGACG	RpoN overexpression
GSU0364-06	GCCAGGGATGCGATCAG	Primer extension
GSU0420-04	AAGCTTTTTCTTGTCCTTTG	Primer extension
GSU0777-04	CTGCCTTCGTGAAATACC	Primer extension
GSU0938-06	GGTCAGGGAAAGAATCTTC	Primer extension
GSU1836-04	CTTCATCCAGCTTGAACG	Primer extension
GSU2005-02	CGATCTTTACGTTGGCTGC	Primer extension
GSU2302-04	CGAAAGAGAAAATCCAGAG	Primer extension
GSU2490-02	GCTACTTGCCACCCCTTG	Primer extension
GSU2751-02	GAGAACGAGAACGAACTGG	Primer extension
GSU2806-08	GGTTGTGTCGTAATAATC	Primer extension
GSU3046-02	CGTCTGACATGAGAGTTTC	Primer extension
GSU3206-06	GAGCCTTCATTTCCTCAG	Primer extension
pGEXrpoNEcoRIfor	GGGAATTCGCCATTGAAATGCGCCAAC	RpoN overexpression for antibody production
pGEXrpoNXhoIrev	GGCTCGAGTCAAAAATGACGTTTGCG	RpoN overexpression for antibody production
